# Association between Circulating Antioxidants and Longevity: Insight from Mendelian Randomization Study

**DOI:** 10.1155/2022/4012603

**Published:** 2022-01-29

**Authors:** Zhimin Yu, Fangfang Zhang, Chengkai Xu, Yanggan Wang

**Affiliations:** ^1^Department of Internal Medicine, Zhongnan Hospital of Wuhan University, Wuhan 430071, China; ^2^Department of Cardiology, Taihe Hospital, Hubei University of Medicine, Shiyan, 442000 Hubei, China; ^3^Department of Pediatrics, Taihe Hospital, Hubei University of Medicine, Shiyan, 442000 Hubei, China; ^4^Department of Internal Medicine, Zhongnan Hospital of Wuhan University, Wuhan University, Wuhan 430071, China; ^5^Medical Research Institute of Wuhan University, Wuhan University, Wuhan 430071, China

## Abstract

**Background:**

Antioxidants attracted long-standing attention as promising preventive agents worldwide. Previous observational studies have reported that circulating antioxidants are associated with reduced mortality; however, randomized clinical trials indicate neutral or harmful impacts. The association of long-term circulating antioxidant exposure with longevity is still unclear.

**Objectives:**

We aim to determine whether long-term circulating antioxidant exposure is causally associated with longevity in the general population using the two-sample Mendelian randomization (MR) design.

**Methods:**

Genetic instruments for circulating antioxidants (ascorbate, lycopene, selenium, beta-carotene, and retinol) and antioxidant metabolites (ascorbate, alpha-tocopherol, gamma-tocopherol, and retinol) were identified from the largest up-to-date genome-wide association studies (GWASs). Summary statistics of these instruments with individual survival to the 90^th^ vs. 60^th^ percentile age (11,262 cases and 25,483 controls) and parental lifespan (*N* = 1,012,240 individuals) were extracted. The causal effect was estimated using the inverse-variance weighted method in the main analysis and complemented by multiple sensitivity analyses to test the robustness of results.

**Results:**

We found that genetically determined higher concentration of circulating retinol (vitamin A) metabolite was casually associated with a higher odds of longevity (OR, 1.07; 95% CI, 1.02–1.13; *P* < 0.01) and increased parental lifespan (lifespan years per 10-fold increase: 0.17; 95% CI, 0.07–0.27; *P* < 0.01). Present evidence did not support a causal impact of circulating ascorbate (vitamin C), tocopherol (vitamin E), lycopene, selenium or beta-carotene on life expectancy. No evidence was identified to show the pleiotropic effects had biased the results.

**Conclusions:**

Long-term higher exposure to retinol metabolite is causally associated with longevity in the general population. Future MR analyses could assess the current findings further by utilizing additional genetic variants and greater samples from large-scale GWASs.

## 1. Introduction

Human longevity is a consequence of the complex interplay between inherited genetic factors and environmental influences [1]. Despite the past two centuries having witnessed the significant increase of life expectancy in the western countries, our knowledge concerning factors that influence longevity is still scarce [2]. It is acknowledged that the heritability of lifespan was consistently low in a variety of populations (12%-25%) [3]. In other words, environmental factors play key roles in ageing process and longevity. Seeking potential environmental determinants of longevity will assist in informing public health strategies and promoting overall health in populations.

Oxidative stress denotes the imbalance between formation of pro-oxidative factors, reactive oxygen species (ROS), and antioxidants, resulting in disturbance of redox signaling and molecular damage [4]. Oxidative modification and damage to cellular DNA, proteins, or lipids due to overproduction of free radicals have been associated with the ageing process [5]. Antioxidants can mitigate oxidative stress and maintain the stable biological redox status through scavenging free radicals. From this prospective, supplementing antioxidant appears to be a promising strategy for slowing down aging and to prolong lifespan [6]. In line with this hypothesis, a large number of observational cohort studies provided evidence that higher dietary or circulating antioxidants (vitamin A, vitamin C, vitamin E, lycopene, selenium, and beta-carotene) were associated with lower risk of all-cause mortality [7–14]. However, making causal inferences from these observational studies is impossible, since observational studies are susceptible to residual confounding and reverse causation bias.

In contrast to the findings from these observational studies, many interventional studies suggest neutral [15–18] or detrimental effects [19, 20] on cardiovascular disease and mortality for antioxidant supplements. For example, a pooled analysis of randomized clinical trials (RCTs) failed to show that adequate vitamin E intake alone can lower the risk of all-cause mortality [18]. Some meta-analyses of RCTs even indicated that individuals supplemented with antioxidants such as beta-carotene, vitamin A, and vitamin E had increased risk of mortality [19, 20]. Conclusions from RCTs can be limited by duration, dosage, and timing of antioxidant supplements [21]. The null findings from RCTs might be a consequence of inadequate therapy duration, since it is unrealistic to reverse the influence of several decades of oxidative stress via only a few years of antioxidant therapy [21]. Whether long-term antioxidant exposure can improve life expectancy in the general population still remains largely unclear.

By using genetic variants as instrumental variables, Mendelian randomization (MR) analysis can infer the causal effects of exposures (circulating antioxidants) on a clinical outcome (longevity) [22]. This method largely avoids residual confounding and reverse causation bias inherent in observational studies, since genetic variants are randomly allocated during meiosis and remain stable throughout the whole life. Meanwhile, MR design is of great value in assessing the lifelong effect of exposure on clinical outcomes. We therefore conducted a two-sample MR analysis to determine whether long-term exposure to circulating antioxidants or its metabolites are causally associated with longevity outcome. We also evaluated its impact on established risk factors of longevity (e.g., type 2 diabetes, hypertension, and smoking initiation) as they may provide mechanistic insights.

## 2. Methods

### 2.1. Two-Sample MR Design

MR relies on three principle assumptions to provide unbiased estimates [23]: (1) genetic instrument should be strongly related to exposure, (2) genetic instrument should be independent of any possible known confounders, and (3) genetic instrument exerts impact on outcomes entirely through the exposure of interest (Supplemental Figure S1). Summary-level statistics from publicly available genome-wide association study (GWAS) were used in this MR study.

**Figure 1:**
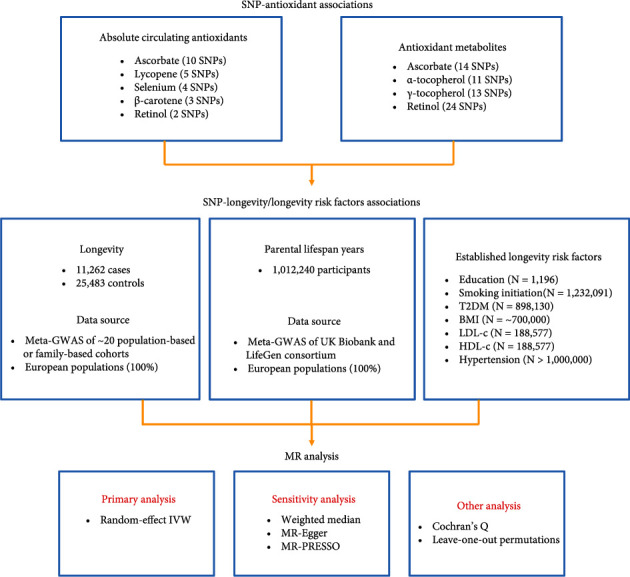
A flow chart detailing data sources and study design for the MR study. Abbreviations: BMI: body mass index; GWAS: genome-wide association study; HDL-c: high-density lipoprotein cholesterol; IVW: inverse-variance weighted; LDL-c: low-density lipoprotein cholesterol; SNPs: single-nucleotide polymorphisms; T2DM: type 2 diabetes mellitus.

Our MR study included six main circulating antioxidants: vitamin C (ascorbate), vitamin E (***α***- and ***γ***-tocopherol are the predominant forms of vitamin E in humans), lycopene, selenium, ***β***-carotene, and retinol (the major biologically active form of vitamin A). Both absolute antioxidants and antioxidant metabolites in the blood were used to infer causality for longevity. In the analyses of absolute antioxidants on longevity, genetic instruments for absolute circulating levels of ascorbate, lycopene, selenium, ***β***-carotene, and retinol were obtained; for antioxidant metabolites, genetic instruments for relative concentrations of ascorbate, ***α***-tocopherol, ***γ***-tocopherol, and retinol were used. The flow chart of the current study is presented in Figure 1.

### 2.2. Data Sources for Absolute Circulating Antioxidants

The largest up-to-date GWAS primarily performed on participants of European descent was used to extract instrumental variables. Summary statistics for blood and toenail selenium concentration are all available, and both have been validated as biomarkers of selenium exposure [24, 25]. Toenail selenium can reflect a longer period of exposure than blood selenium. A GWAS of toenail selenium, which included four US cohorts, evaluated the associations of genetic variants with both toenail and blood selenium concentrations; we therefore chose this GWAS as the data source for selenium exposure [25]. Data sources for included instrumental variables are summarized in Table 1.

Briefly, instrumental variables for circulating ascorbic acid concentration were derived from the up-to-date meta-analysis of GWAS, comprising up to 52,018 individuals of European ancestry [26]. 11 leading single nucleotide polymorphisms (SNPs) that were significantly related to circulating ascorbic acid level at the genome-wide significance level (*P* < 5.0 × 10^−8^) were identified. One SNP, rs174547, located in the *FADS1* gene was excluded due to its pleiotropic effects, leaving 10 SNPs for our analysis [26]. Instrumental variables for circulating lycopene were obtained from a GWAS conducted on 441 old order Amish adults who consumed a controlled diet [27]. A total of five SNPs associated with the circulating lycopene level at the genome-wide (*P* < 5.0 × 10^−8^) or subgenome-wide (*P* < 1.0 × 10^−6^) significance level were identified as genetic instruments [27].

Instrumental variables for selenium concentration were derived from a sample size-weighted meta-analysis comprising 4,162 participants of European ancestry [25]. Four SNPs were identified at the genome-wide significance level (*P* < 1.0 × 10^−8^) and linkage disequilibrium (LD) < 0.3. Instrumental variables for circulating *β*-carotene were extracted from a GWAS performed on the Nurses' Health Study including 2,344 women of European ancestry. Three leading SNPs associated with the plasma *β*-carotene level at the genome-wide significance level (*P* < 5.0 × 10^−8^) were obtained [28]. Instrumental variables for circulating retinol concentration were derived from a GWAS based on 5006 Caucasian participants drawn from two cohorts of men [29]. Two SNPs associated with the serum retinol level at the genome-wide significance level (*P* < 5.0 × 10^−8^) were selected [29]. The variance of circulating antioxidant concentrations explained by instrumental variables ranged from 1.87% for ascorbate to 30.1% for lycopene (Table 1).

### 2.3. Data Sources for Circulating Antioxidant Metabolites

SNPs associated with blood antioxidant metabolites of ascorbate (*N* = 14), ***α***-tocopherol (*N* = 11), and ***γ***-tocopherol (*N* = 13) at the suggestive genome-wide significance level (*P* < 1.0 × 10^−5^) came from a publicly available GWAS based on two European cohort studies [30]. This GWAS reported genome-wide associations of metabolic locus with more than 400 metabolites in human blood from 7,824 individuals of European ancestry. SNPs associated with the blood retinol metabolite level (*N* = 24) at the suggestive genome-wide significance level (*P* < 1.0 × 10^−5^) were derived from a recently published GWAS comprising 1,957 European participants [31]. The variance of circulating antioxidant metabolites explained by genetic instruments varied from 3.3% for ***α***-tocopherol to 18.6% for ascorbate (Table 1).

### 2.4. Data Sources for Longevity Outcome

Summary statistics for antioxidant-associated SNPs with individual longevity were extracted from a publicly available GWAS, which recruited individuals of European ancestry from ~20 population-based or family-based cohorts [32]. This GWAS discriminated cases from controls based on ages that correspond to different survival percentiles. Therefore, the influence of heterogeneity between cases and controls can be mitigated [32]. Genetic associations of SNPs with odds of individuals surviving to the 90^th^ percentile age (*N* = 11,262) vs. the 60^th^ percentile age (*N* = 25,483) according to the country, sex, and birth cohort-specific life table were extracted (*the 90th vs. 60th percentile age dataset*). The majority of included cohorts have less controls than cases, as many participants enrolled were already relatively old at the beginning of follow-up [32].

Summary statistics for life expectancy were additionally obtained from a meta-GWAS of the UK Biobank and LifeGen consortium [33]. This GWAS investigated the Cox proportional hazard model-quantified parental lifespan from a cohort of 1,012,240 parents of European descent. Parental lifespan can be served as a proxy outcome for individual longevity in genetic association study, as offspring shares 50% of its genome with each parent. The effect estimates extracted from this GWAS may be multiplied by ten to reflect the absolute changes in lifespan years [33].

### 2.5. Mendelian Randomization Analysis

In the primary analyses, we applied the inverse-variance weighted (IVW) approach under a multiplicative random-effect model to estimate the causal effects [34]. The Wald ratio estimates from each individual SNP were pooled to reflect the overall causal effect of the antioxidant on longevity. This approach can provide the highest precision and unbiased causal estimate when invalid genetic instruments were absent [35].

To test the robustness of causal inference and check for pleiotropy, we conducted sensitivity analyses using the weighted median [36], MR-Egger method [37], and the MR pleiotropy residual sum and outlier (MR-PRESSO) test [38]. The weighted median method gives reliable estimates if over 50% of the weight in the analysis derives from valid genetic instruments [36]. Potential directional pleiotropy was assessed with the MR-Egger method, where the intercept term constrained to zero suggested the absence of directional pleiotropy [37]. The MR-Egger regression method renders pleiotropy-corrected causal effect estimates, though having a relative lower precision than weighted median method. The MR-PRESSO approach was used to identify outlier instrument variables that were potentially horizontally pleiotropic. Similar to the MR-Egger method, this approach provides corrected causal estimates after removal of outliers [38]. In addition, we evaluated the heterogeneity between instrument variables by using Cochran's *Q* test and *P* value [35]. Finally, “leave-one-out” sensitivity analysis was performed to check if a single SNP had undue effect on the overall estimated effect size.

Genetic variants may influence longevity outcome through potential confounders or alternative pathways independent of their impacts on circulating antioxidant, namely, horizontal pleiotropy [39]. To examine whether this could bias the MR results, we assessed the effects of included SNPs on established risk factors for longevity. Seven risk factors which had been demonstrated to be causally associated with longevity from a previous MR study were considered: years of education, smoking initiation, type 2 diabetes, body mass index (BMI), low-density lipoprotein cholesterol (LDL-c), high-density lipoprotein cholesterol (HDL-c), and hypertension [40]. Details of included GWAS data sets for these risk factors are presented in Supplemental Table S7. The majority of include participants were of European descent, and sample sizes ranged from 188,577 for HDL-c to 1,232,091 for smoking initiation [40]. We assessed the effects of absolute circulating antioxidants and metabolites on these seven risk factors using the IVW approach.

To determine the power of our MR results, post hoc power calculations were conducted for the IVW analyses using an online calculator (https://sb452.shinyapps.io/power/) [41]. *F* statistic reflecting the exposure variance explained by instrumental variable was used to assess the instrument strength [42]. The *F* statistic was either extracted from original exposure GWAS or calculated based on reported *R*^2^ values and sample sizes when these were not reported (*F* = *R*^2^(*n* − *k* − 1)/*k*(1 − *R*^2^); *R*^2^ represents the variance of exposure explained by instrumental variables, *n* represents the sample size, and *k* represents the number of instrumental variables). *F* statistic above 10 suggests that the possibility of weak instrument bias is small [42].

In the longevity analysis, results are reported as odds ratios (ORs) with corresponding 95% confidence intervals (CIs), reflecting one unit change in absolute concentrations of antioxidants on natural log-transformed levels (*β*-carotene, retinol, and selenium), mg/dl (lycopene), mmol/l (ascorbate), or a 10-fold change for metabolite concentrations. In complementary analysis of parental lifespan, results are reported as changes in lifespan years with corresponding 95% CIs. A *P* value < 0.05 was considered statistically significant. All statistical analyses were performed using R (Version 3.6.2) using the “TwoSampleMR” [35] and “MR-PRESSO” [38] packages.

### 2.6. Ethics

Publicly available summary-level data were utilized in the MR analysis; therefore, no additional ethical approval was required. Ethical approval and informed consent for each included study can be identified in each original publication. All involved studies were in accordance with the Declaration of Helsinki.

## 3. Results

Genetic instruments associated with circulating antioxidants and their metabolites are detailed in Table 1. There was no sample overlap between the GWAS of exposure and the GWAS of longevity. The cohort characteristics and exclusion criteria for individual longevity GWAS and parental lifespan GWAS are presented in Supplemental Tables S2 and S3, respectively. Supplemental Tables S4 and S5 show the effect estimates for the associations of selected SNPs with circulating antioxidants (***β***_SNP-antioxidants_) and with longevity outcomes (*β*_SNP-longevity_), respectively. *F* statistic for each used SNPs was >10, minimizing the potential weak instrument bias. The bioavailability and mode of action of these antioxidants are summarized in Table 2.

### 3.1. Absolute Circulating Antioxidants and Longevity

**Figure 2:**
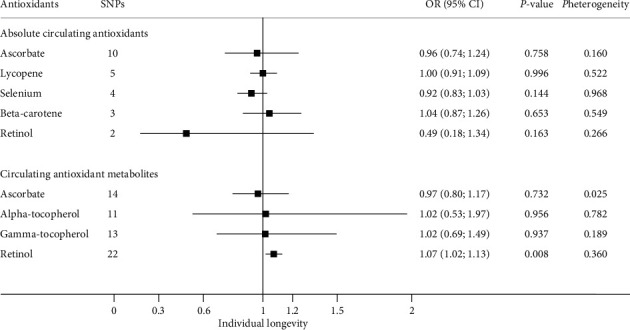
Odds ratios for relationships between genetically predicted circulating antioxidants and longevity (*n* = 36,745). The ORs for absolute antioxidants represent the effect of per unit increase in ln-transformed selenium, ***β***-carotene, and retinol values, 1 mmol/l ascorbate and 1 mg/dl lycopene, on longevity. For antioxidant metabolites, ORs represent the effect of per 10-fold increase in the antioxidant metabolite's level on longevity. All estimates were calculated based on random-effect IVW method. The *P*_heterogeneity_ values derived from the Cochran's *Q* statistics were used to reflect heterogeneity between the SNP-specific estimates. Abbreviations: CI: confidence interval; IVW: inverse-variance weighted; OR: odds ratio; SNP: single nucleotide polymorphism.

**Figure 3:**
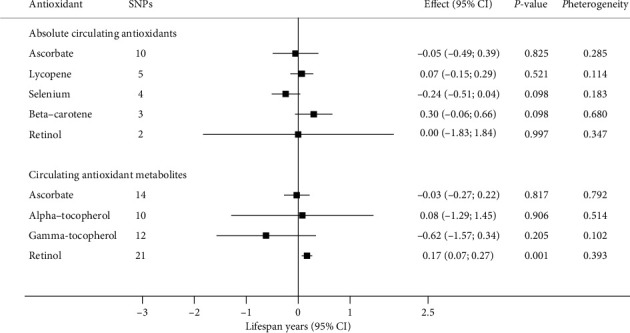
MR estimates for relationships between genetically predicted circulating antioxidants and lifespan years (*n* = 1,012,240). The effect estimates for absolute antioxidants represent the change in lifespan years per unit increase in ln-transformed selenium, ***β***-carotene, and retinol values, 1 mmol/l ascorbate and 1 mg/dl lycopene. For antioxidant metabolites, the estimates are expressed as change in lifespan years per 10-fold increase in antioxidant metabolite's level. All estimates were calculated based on random-effect IVW method. The *P*_heterogeneity_ values derived from Cochran's *Q* statistics were used to reflect heterogeneity between the SNP-specific estimates. Abbreviations: CI: confidence interval; IVW: inverse-variance weighted; MR: Mendelian randomization; SNP: single nucleotide polymorphism.

Our principal results obtained from the IVW method showed that there was no significant association of absolute circulating antioxidant concentration with longevity (Figures 2 and 3). The OR for survival to the 90th vs. 60th percentile age per unit increase of antioxidant concentration was 0.96 for ascorbate (per 1 mmol/l increase, 95% CI: 0.74–1.24; *P* = 0.758), 1.00 for lycopene (per 1 mg/dl increase, 95% CI: 0.91–1.09; *P* = 0.996), 0.92 for selenium (per ln-transformed increase, 95% CI: 0.83–1.03; *P* = 0.144), 1.04 for ***β***-carotene (per ln-transformed increase, 95% CI: 0.87–1.26; *P* = 0.653), and 0.49 for retinol (per ln-transformed increase, 95% CI: 0.18–1.34; *P* = 0.163) (Figure 2). The association of a unit increase in genetically predicted absolute antioxidant concentration with lifespan years was -0.05 for ascorbate (per 1 mmol/l increase, 95% CI: -0.49–0.39; *P* = 0.825), 0.07 for lycopene (per 1 mg/dl increase, 95% CI: -0.15–0.29; *P* = 0.521), -0.24 for selenium (per ln-transformed increase, 95% CI: -0.51–0.04; *P* = 0.098), 0.30 for ***β***-carotene (per ln-transformed increase, 95% CI: -0.06–0.66; *P* = 0.098), and 0.003 for retinol (per ln-transformed increase, 95% CI: -1.83–1.84; *P* = 0.997) (Figure 3).

Sensitivity analyses on all antioxidants except for retinol were performed using the MR-Egger, weighted median, and MR-PRESSO approaches. No significant associations were obtained from either of the MR-Egger or weighted median analysis.

No significant heterogeneity between individual SNPs was observed with Cochran's *Q* test (all *P* values for heterogeneity > 0.05, Table 3, Supplemental Table S6). Furthermore, MR-Egger regression did not support the presence of potential pleiotropy as the intercepts constrained to zero (*P*_Intercept_ > 0.05). MR-PRESSO analysis did not identify any potential outlier SNPs (Table 3, Supplemental Table S6). In addition, similar null associations were obtained after omitting each SNP in turn in the leave-one-out analysis, demonstrating that no single SNP was having undue effect on the overall estimated effect size (Supplemental Figures S2-5).

### 3.2. Circulating Antioxidant Metabolites and Longevity

Similar with the findings from analyses of absolute antioxidants, there was no significant difference in longevity or lifespan years according to antioxidant metabolite concentrations of ***α***-tocopherol, ***γ***-tocopherol, and ascorbate. However, significant association between retinol metabolite concentration and longevity was observed (Figures 2 and 3). Specifically, a 10-fold increase in retinol metabolite was causally associated with 7% higher odds of longevity (OR 1.07; 95% CI: 1.02–1.13; *P* < 0.01, Figure 2). Similarly, significantly higher lifespan years were also observed for genetically predicted higher retinol metabolite level (lifespan years per 10-fold increase: 0.17; 95% CI: 0.07–0.27; *P* < 0.01, Figure 3).

In sensitivity analyses of the associations of antioxidant metabolites with longevity, no evidence of horizontal pleiotropy was indicated (*P*_Intercept_ in MR-Egger regression was >0.05; Table 3, Supplemental Table S6). In addition, no significant heterogeneity between SNPs was suggested, except for ascorbate where moderate heterogeneity was presented in the analysis of *the 90th vs. 60th percentile age dataset* (*P* = 0.025). However, this heterogeneity was unlikely to influence the MR estimate, as weighted median approach provided comparable result to the IVW method (Table 3). Although MR-PRESSO detected 1 outlier SNP for ascorbate in the analysis of individual longevity, the conclusion did not alter after removing the outlier. Furthermore, the leave-one-out analysis performed in this dataset found no undue influence of potentially pleiotropic SNPs on the overall causal inference (Supplemental Figures S6-9).

### 3.3. Antioxidant Associations with Longevity Risk Factors

**Figure 4:**
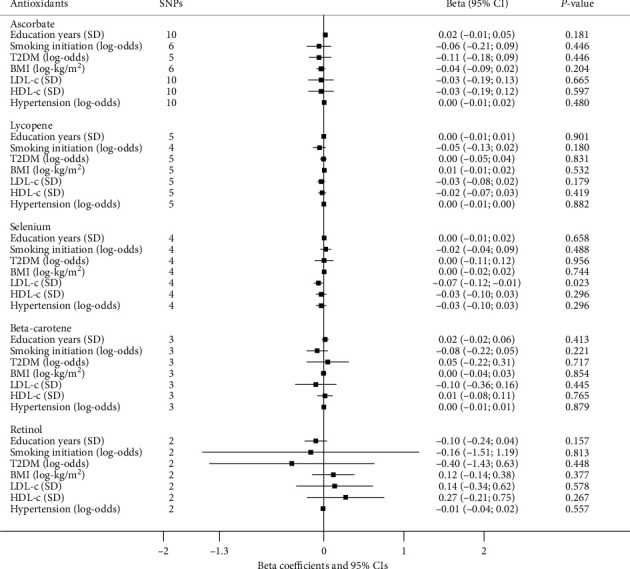
MR results for absolute antioxidant exposure and factors that may determine life expectancy. Results are reported as beta coefficients and 95% CIs showing factor unit differences (in parentheses) per unit increase in ln-transformed ***β***-carotene and retinol values, 1 mmol/l ascorbate and 1 mg/dl lycopene. The random-effect IVW method was applied in the MR analysis. *P* value below 0.05 was highlighted.

**Figure 5:**
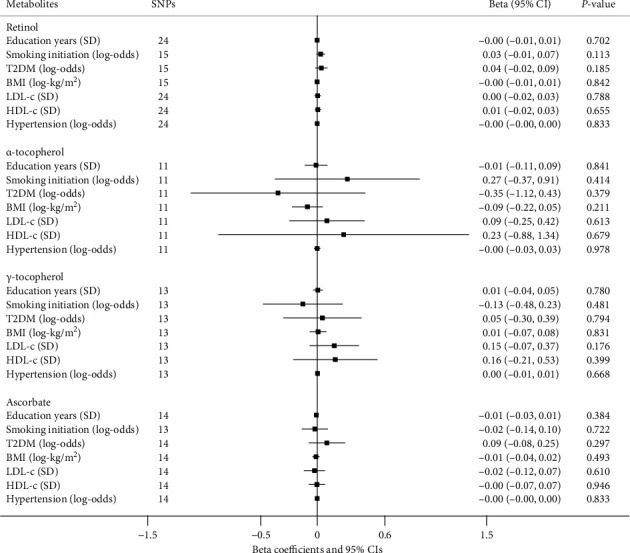
MR results for antioxidant metabolite exposure and factors that may determine life expectancy. Results are reported as beta coefficients and 95% CIs showing factor unit differences (in parentheses) per 10-fold increase in antioxidant metabolites' level. Random-effect IVW method was applied in the MR analysis.

The effects of absolute antioxidant on established longevity risk factors with IVW approach are depicted in Figure 4. In general, there is no clear pattern of correlation between circulating absolute antioxidant exposure and longevity risk factors. The only exception was an inverse association of selenium with LDL-c level (beta: -0.07; 95% CI: -0.12 to -0.01; *P* = 0.023). Nevertheless, this association did not alter the null findings between selenium and longevity outcome. Genetically predicted antioxidant metabolites were not associated with any of these longevity risk factors (all *P* > 0.05, Figure 5).

## 4. Discussion

### 4.1. Principal findings

For the first time, the effect of long-term circulating antioxidant exposure on longevity outcome was assessed using the MR approach. Based on large-scale longevity consortia, our study provided evidence for a causal relationship of higher circulating retinol metabolite with higher odds of being long-lived. However, our study did not support the association of circulating tocopherol, lycopene, selenium, ***β***-carotene, or ascorbate with life expectancy. The null associations were consistent across *the 90th vs. 60th percentile age dataset* and *parental lifespan dataset*, implying that these circulating antioxidants are unlikely to be causal factors for life expectancy. Furthermore, our study indicated an inverse association of circulating selenium with plasma LDL-c level, although its effect on longevity was close to null.

During the past several decades, people consume antioxidants to prevent diseases and promote health worldwide [53]. Controversy still remains across observational studies and RCTs, and no general agreement on single antioxidants should be used as supplements for mortality prevention. It is of great public health significance, since up to 52% of the population take these supplements in 2012 according to the National Health and Nutrition Examination Survey data (1999 to 2012) [54]. Using a genetic approach, this study provides evidence that circulating retinol, but not other antioxidants, is causally related to longevity. The magnitude of the impact of included genetic variants on the antioxidant level was summarized in Supplemental Table S8. Overall, the impacts of these genetic variants are comparable to or within the range of the effects of supplementation in RCTs. However, outcomes obtained from these two methods can be different. It is presumed that the impact of these genetic variants was lifelong, whereas the impact of antioxidant supplementation may merely last for the duration of the trial. A slightly minor exposure throughout the whole life course would exert greater potential biological influence than the temporarily larger impact of supplements, considering aging is a consequence of long-term accumulation of molecular damage. It is noteworthy that our findings did not rule out the favorable roles of fruit and vegetables, which contain numerous micronutrients and fibers besides the abovementioned antioxidants.

### 4.2. Comparisons with other studies

Our study suggested that circulating retinol metabolite was positively related to increased lifespan years, a finding that was similar to those of interventional trials conducted in children, which showed that vitamin A supplementation was associated with 12% reduction in mortality [55, 56]. Previous studies have suggested that vitamin A deficiency increases the risk of a range of diseases, including respiratory disease, diarrhoea, measles, and vision problem [57]. All these aforementioned diseases have been considered the leading cause of mortality among children [58]. On the other hand, RCTs performed in children aged six months to five years support the beneficial effect of vitamin A supplementation on morbidity and mortality [56]. Based on these evidences, vitamin A supplementation has been continually recommended by the World Health Organization for children aged 6 to 59 months. However, current evidence does not support the beneficial effect of vitamin A supplementation among neonates and adults in reducing mortality, albeit the certainty of evidence was low [59, 60]. Furthermore, prior MR studies found no effect of higher blood vitamin A concentration on risk of coronary heart disease [61], ovarian cancer [62], and Alzheimer disease [63]. The longevity outcome may be considered in relation to all these effects over the life course. The beneficial effect of circulating retinol on longevity observed in our study might be explained, at least in part, by the significant reduction in all-cause mortality during children's period, considering that the assessed effect of retinol was lifelong in the MR study. In addition, the null finding for absolute retinol concentration on longevity might be a consequence of insufficient power, given the small variance of absolute retinol concentration explained by only two SNPs (2.3%). This is further supported by the wide confidence intervals for the effect estimates. On the other hand, genetic instruments for retinol metabolite explained greater proportion of phenotypic variance (4.8%) showed a significant association with longevity.

The mechanisms underlying the beneficial effect of retinol on longevity are complicated, and several explanations exist. It is presumed that oxidative stress plays import roles in aging process and various age-related disorders [6]. Meanwhile, a decrease in lipophilic antioxidants was observed in clinical and animal studies during aging [64]. The lipophilicity of dietary antioxidants may play a critical role in the aging process, since it enables the compound to accumulate within the organism for extended effects. As a lipophilic antioxidant, plasma retinol has been considered as an important micronutrient in guaranteeing extreme longevity [65]. This could be partially attributed to its antioxidant property (Table 2). However, many other properties of this antioxidant that were observed in the biological systems may also justify its beneficial effects during life. For example, retinol has an essential role in maintaining normal functions of neutrophils, macrophages, and natural killer cells during infection [66]. Retinol is also needed for adaptive immunity, and its deficiency diminished antibody-mediated responses [66]. Moreover, retinol and vitamin A derivatives can affect cell differentiation, proliferation, and apoptosis and are inversely associated with several types of cancers [67]. Further investigations are still required to determine to what extent these actions are affected by the antioxidant activity of retinol or by other potential mechanisms.

Previous studies reported inconsistent associations between circulating vitamin E and risk of all-cause mortality. For instance, in a meta-analysis which included 37,199 participants from six prospective cohorts, a higher circulating ***α***-tocopherol level was significantly associated with a lower risk of all-cause mortality [68]. However, other meta-analyses based on RCTs indicated reverse conclusions, especially for the highest supplement dosage (≥400 IU/d) [20, 69–71]. Significant heterogeneity between observational studies was observed for the effect estimates in the meta-analysis. This implies that results from these observational studies can be biased by potential confounders, such as physical activity, BMI, educational status, lipid profiles, and alcohol consumption. Despite the relatively consistent evidence of observational studies, it is still challenging to clarify whether circulating ***α***-tocopherol causally affects life expectancy. This can be attributed to the biases such as residual confounding that undermine the validity in observational studies, even when the confounding factors had been adjusted. Meanwhile, a single detection of the serum *α*-tocopherol level at baseline might not reflect the long-term status of vitamin E, since vitamin E and its metabolite levels could have altered over time [14]. On the other hand, many RCTs were conducted with very short follow-up period (i.e., <2 years) [60], which would significantly hinder the evaluation of long-term effect of vitamin E exposure on longevity outcome. In this setting, the MR study using genetic instruments as proxies can reflect lifelong exposure to a certain extent with a higher tocopherol concentration and can largely avoid biases in observational studies. However, our findings did not support the beneficial or detrimental effects of higher serum tocopherol on longevity. These results were robust in a wide variety of sensitivity analyses with no indication of violation of the MR assumptions.

Our study also does not support the association of circulating lycopene with longevity with no evidence of violation of the MR principals (Table 3). Moreover, the association was consistent with several observational studies on circulating lycopene and all-cause mortality [72, 73], despite the fact that these observational studies had relatively small sample size, and circulating lycopene concentration was measured at a single time-point only.

Previous observational studies concerning selenium, vitamin C, and ***β***-carotene on risk of all-cause mortality also yielded controversial results. One recent meta-analysis which included 41 prospective observational studies based on the general population (*n* = 507,251) indicated inverse associations of circulating selenium, vitamin C, and ***β***-carotene concentrations with all-cause mortality [68]. However, significant between-study heterogeneity was noted in the analyses for selenium (*I*^2^ = 84.4%, *P* for heterogeneity < 0.01) and vitamin C (*I*^2^ = 69.2%, *P* for heterogeneity <0.05), and publication bias was also suggested [68]. Another pooled analysis of 53 RCTs showed a significantly increased risk of mortality for *β*-carotene supplement, whereas no effect was indicated for selenium or vitamin C supplement [69]. In these interventional trails, antioxidant supplement may not be linearly associated with the circulating concentration of antioxidant, since it can be influenced by various factors such as absorption effectiveness, age, smoking, comorbidities, and physical activity. Considering the null associations of circulating selenium, vitamin C, and *β*-carotene with longevity detected by our MR analyses, and no evidence of violating the MR assumptions, the inconsistent results from previous observational trials raised concern about confounding. Fruit and vegetables are the primary sources of these dietary antioxidants; meanwhile, they are both abundant in fibers and polyphenols, and both have been linked with better health status [74]. Therefore, higher circulating concentrations of these antioxidants might imply higher intake of fruit and vegetables [74], which would represent the total dietary patterns instead of a single micronutrient measure.

### 4.3. Strengths and Limitations

A major strength of the present study was the use of two-sample MR design, which overcomes the short duration of antioxidant exposure in RCTs and minimizes potential confounding bias in observational investigations and adds to the body of knowledge on dietary antioxidants and longevity. Another strength was that we used two European datasets with large sample size (>1 million) for SNP-longevity associations in this MR analysis. Thus, we had adequate statistical power to identify even modest causal associations for most analyses (Supplemental Table S1). The results obtained from two longevity datasets were largely consistent with no indication of heterogeneity generally, and model violations (mainly from pleiotropy) were explored by various methodologies also increased the robustness of our findings, despite the limited number of strong instrument variables used in partial analysis. Furthermore, no sample overlap in the exposure GWASs and longevity GWASs has lowered the type 1 error rate (Table1).

Our study also has several limitations which merit further consideration. First, individuals had to survive to a certain age to be recruited in the longevity GWASs. As a consequence, individuals with early death were missing from the control group, and the true effect might be underestimated if those individuals have more unfavorable risk factors. Second, we are unable to evaluate nonlinearity in the associations of circulating antioxidants with life expectancy that have previously been suggested, in particular for circulating lycopene [68]. Future investigations utilizing individual-level data to fully clarify the potential dose-response associations are still needed. Third, higher circulating lycopene, selenium, ***β***-carotene, and vitamin C concentrations might still be beneficial in discriminatingly selected populations who have risk factors at baseline or under increased oxidative stress status. For instance, protective effects of circulating ***β***-carotene and selenium on mortality have been demonstrated to be more obvious in individuals aged above 60 years when compared with younger individuals [68]. However, our study could not evaluate the effect of these antioxidants on longevity in subgroups with different risk factors or lacking certain kind of nutrients where antioxidant supplement appears to be more beneficial. Finally, our findings are only generalizable to individuals of European descent.

## 5. Conclusions

In conclusion, our study supports the hypothesis that higher circulating retinol concentration is causally associated with increased life expectancy. Current evidence casts doubt over the beneficial or detrimental effect of higher circulating lycopene, selenium, *β*-carotene, or vitamin C concentrations in longevity. Supplementing these antioxidants in the general population without a definite deficiency will have little benefit for life expectancy. Future MR analyses designed to expand on the current findings by exploiting larger number of SNPs to proxy circulating antioxidants based on large-scale GWASs are still warranted.

## Figures and Tables

**Table 1:** Description of data sources of the genetic instruments used for circulating antioxidants in the Mendelian randomization study.

Antioxidant	Sample size	No. of SNPs	Unit	Ancestry	*P* value	Variance (%)^†^	Overlap^§^	PMID
Absolute circulating antioxidants								
Ascorbate	15,087	10	*μ*mol/l	European	5*E*-08	1.87%	None	33203707
Lycopene	441	5	*μ*g/dl	European	5*E*-08	30.1%	None	26861389
Selenium	4,162	4	*μ*g/g in natural log-transformed scale	Primarily European∗	5*E*-08	5.9%	None	25343990
*β*-Carotene	2,344	3	*μ*g/l in natural log-transformed scale	European	5*E*-08	9.0%	None	23134893
Retinol	5,006	2	*μ*g/l in natural log-transformed scale	European	5*E*-08	2.3%	None	21878437
Circulating antioxidant metabolites								
Ascorbate	2,063	14	Log10-transformed metabolites	European	1*E*-05	18.6%	None	24816252
*α*-Tocopherol	7,276	11	Log10-transformed metabolites	European	1*E*-05	3.3%	None	24816252
*γ*-Tocopherol	5,822	13	Log10-transformed metabolites	European	1*E*-05	15.0%	None	24816252
Retinol	1,957	24	Log10-transformed metabolites	European	1*E*-05	4.8%	None	28263315

^∗^Study population contains both Europeans and African-American participants. ^†^Explained variance for circulating antioxidant metabolites were as reported in GWASs or calculated using the formula of $R^{2}={\left(\beta \times \sqrt{2\times \text{MAF}\left(1-\text{MAF}\right)}\right)}^{2}$ assuming no genetic interactions, where MAF denotes the minor allele frequency and *β* denotes the effect of SNPs on the antioxidant metabolites. ^§^The estimated overlap of the longevity GWAS with the exposure GWASs. Abbreviation: SNPs: single-nucleotide polymorphisms.

**Table 2:** Bioavailability and mode of action of the antioxidants included in this MR study.

Antioxidants	Bioavailability	Mode of action
*β*-Carotene	4%-14% [43]	Antioxidant activity, regulate detoxification enzymes, immune response and hormone metabolism, antibacterial and antiviral effects, anticancer property [44]
Lycopene	0.1%-1.5% [43]	Maintain redox homeostasis, enhance autophagy, anti-inflammation, reduce lipid peroxidation and LDL-c, inhibit proliferation of neoplastic cells [44]
Selenium	55%-65% [45]	Maintain redox homeostasis, maintain the physiological functions of brain, anti-inflammatory and antiviral properties, participate in thyroid hormone metabolism, cancer prevention [46]
Vitamin A	75%-100% [47]	Maintain normal functions of visual system and reproduction, anti-inflammation, modulate immunity, promote cell differentiation, regulate cell growth, maintain epithelial integrity, antithrombosis, cancer prevention [48]
Vitamin C	49%-90% [49]	Antioxidant function, increase endothelium-dependent vasodilatation, inhibit lipid peroxidation, increase the absorption of iron, participate in energy-yielding metabolism and collagen synthesis [50]
Vitamin E	50%-80% [51]	Antioxidant function, anticancer effects, anti-inflammation, inhibit cell proliferation, antiangiogenesis, immune modulation, inhibit HMG-CoA reductase enzyme [52]

Abbreviations: LDL-c: low-density lipoprotein cholesterol; HMG-CoA: 3-hydroxy-3-methylglutaryl-coenzyme A.

**Table 3:** IVW findings and sensitivity analyses for genetically predicted per unit increase in circulating antioxidants and individual longevity outcome.

Antioxidants	IVW/Wald ratio			Weighted median		MR-Egger			MR-PRESSO		
	OR (95% CI)	*P* value	*P* value for heterogeneity	OR (95% CI)	*P* value	OR (95% CI)	*P* value	Intercept (*P* value)	No. of outliers	OR (95% CI)	*P* value
*Absolute circulating levels*											
Ascorbate (***μ***mol/l)	0.96 (0.74-1.24)	0.758	0.160	1.17 (0.88-1.57)	0.281	1.24 (0.87-1.79)	0.272	-0.024 (0.110)	0	0.96 (0.74-1.24)	0.766
Lycopene (*μ*g/dl)	1.00 (0.91-1.09)	0.996	0.522	0.99 (0.88-1.11)	0.805	0.90 (0.76-1.06)	0.299	0.043 (0.081)	0	1.00 (0.92-1.08)	0.996
Selenium (ln-transformed)	0.92 (0.83-1.03)	0.144	0.968	0.93 (0.82-1.05)	0.229	0.98 (0.69-1.40)	0.931	-0.016 (0.285)	0	0.92 (0.89-0.95)	0.015
*β*-Carotene (ln-transformed)	1.04 (0.87-1.26)	0.653	0.549	1.04 (0.85-1.26)	0.715	0.85 (0.42-1.68)	0.716	0.028 (0.615)	NA	NA	NA
Retinol (ln-transformed)	0.49 (0.18-1.34)	0.163	0.266	NA	NA	NA	NA	NA	NA	NA	NA
*Circulating metabolites*											
*α*-Tocopherol (log10 units)	1.02 (0.53-1.97)	0.955	0.782	1.02 (0.42-2.50)	0.960	0.66 (0.16-2.77)	0.581	0.013 (0.430)	0	1.02 (0.69-1.49)	0.945
*γ*-Tocopherol (log10 units)	1.02 (0.69-1.49)	0.936	0.189	1.09 (0.66-1.79)	0.733	1.30 (0.58-2.90)	0.537	-0.011 (0.507)	0	1.01 (0.69-1.49)	0.938
Retinol (log10 units)	1.07 (1.02-1.13)	0.008	0.360	1.07 (0.99-1.15)	0.050	1.01 (0.87-1.18)	0.877	0.012 (0.512)	0	1.07 (1.02-1.13)	0.015
Ascorbate (log10 units)	0.97 (0.80-1.17)	0.731	0.025	0.80 (0.64-1.00)	0.050	0.96 (0.66-1.40)	0.838	7.04*E*-04 (0.970)	1	0.86 (0.72-1.02)	0.116

Abbreviations: CI: confidence interval; IVW: inverse-variance weighted; MR-PRESSO: MR pleiotropy residual sum and outlier; NA: not available; OR: odds ratio.

## Data Availability

All data described in the article are provided within the article.

## Supplementary Materials

Supplementary Materials Supplemental Table S1: post hoc power calculation for the main IVW analyses on circulating antioxidants and longevity. Supplemental Table S2: cohort characteristics and exclusion criteria for the individual longevity GWAS. Supplemental Table S3: cohort characteristics and exclusion criteria for the parental lifespan GWAS. Supplemental Table S4: effect estimates for the associations of the included instrumental variants with absolute circulating antioxidants and longevity outcome. Supplemental Table S5: effect estimates for the associations of the selected instrumental variants with circulating antioxidant metabolites and longevity outcome. Supplemental Table S6: IVW findings and sensitivity analyses for genetically predicted per unit increase in circulating antioxidants and parental lifespan outcome. Supplemental Table S7: details of GWAS datasets used to test associations of antioxidant-instrumenting SNPs with potential confounders and other determinants of longevity. Supplemental Table S8: comparison of the impacts on circulating antioxidant levels between genetic instruments and antioxidant supplementation. Supplemental Figure S1: three principal assumptions in MR study. Supplemental Figure S2: leave-one-out permutations for the association of absolute circulating ascorbate with longevity. Supplemental Figure S3: leave-one-out permutations for the association of absolute circulating lycopene with longevity. Supplemental Figure S4: leave-one-out permutations for the association of absolute circulating selenium with longevity. Supplemental Figure S5: leave-one-out permutations for the association of absolute circulating beta-carotene with longevity. Supplemental Figure S6: leave-one-out permutations for the association of alpha-tocopherol metabolite with longevity. Supplemental Figure S7: leave-one-out permutations for the association of gamma-tocopherol metabolite with longevity. Supplemental Figure S8: leave-one-out permutations for the association of circulating retinol metabolite with longevity. Supplemental Figure S9: leave-one-out permutations for the association of circulating ascorbate metabolite with longevity.
