# Supplementary material for: Association between Circulating Antioxidants and Longevity: Insight from Mendelian Randomization Study
Source: Biomed Res Int. 2022 Jan 29;2022:4012603. doi: 10.1155/2022/4012603 (PMC8817834; doi:10.1155/2022/4012603)

Online Supporting Material

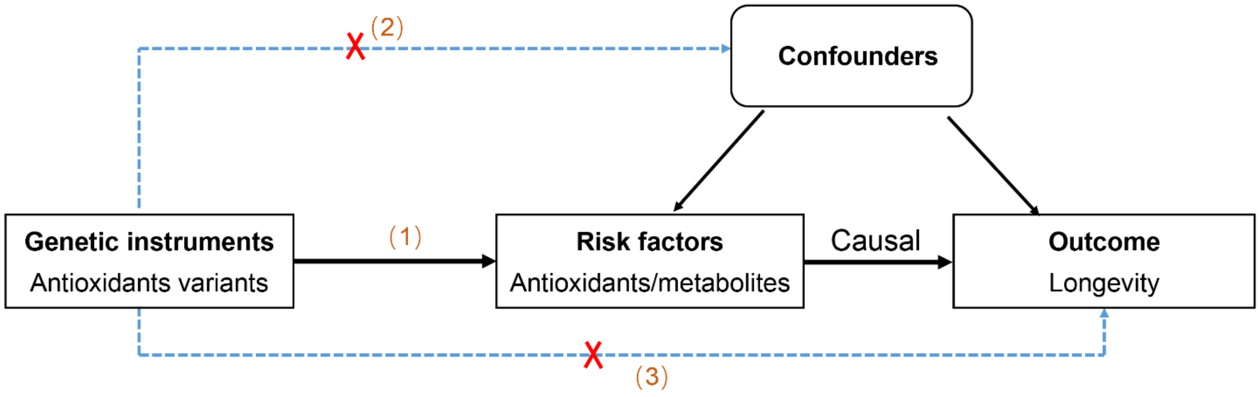

Supplemental Figure S1: Three principal assumptions in MR study: (1) genetic instruments are strongly related to exposure (circulating antioxidants/metabolites); (2) genetic instruments are independent of any possible known confounders; (3) genetic instrument exert impact on outcomes (longevity) entirely through the exposure (circulating antioxidants/metabolites), not through other pathways.

Online Supporting Material

Supplemental Figure S2: Leave-one-out permutations for the association of absolute circulating ascorbate with longevity.

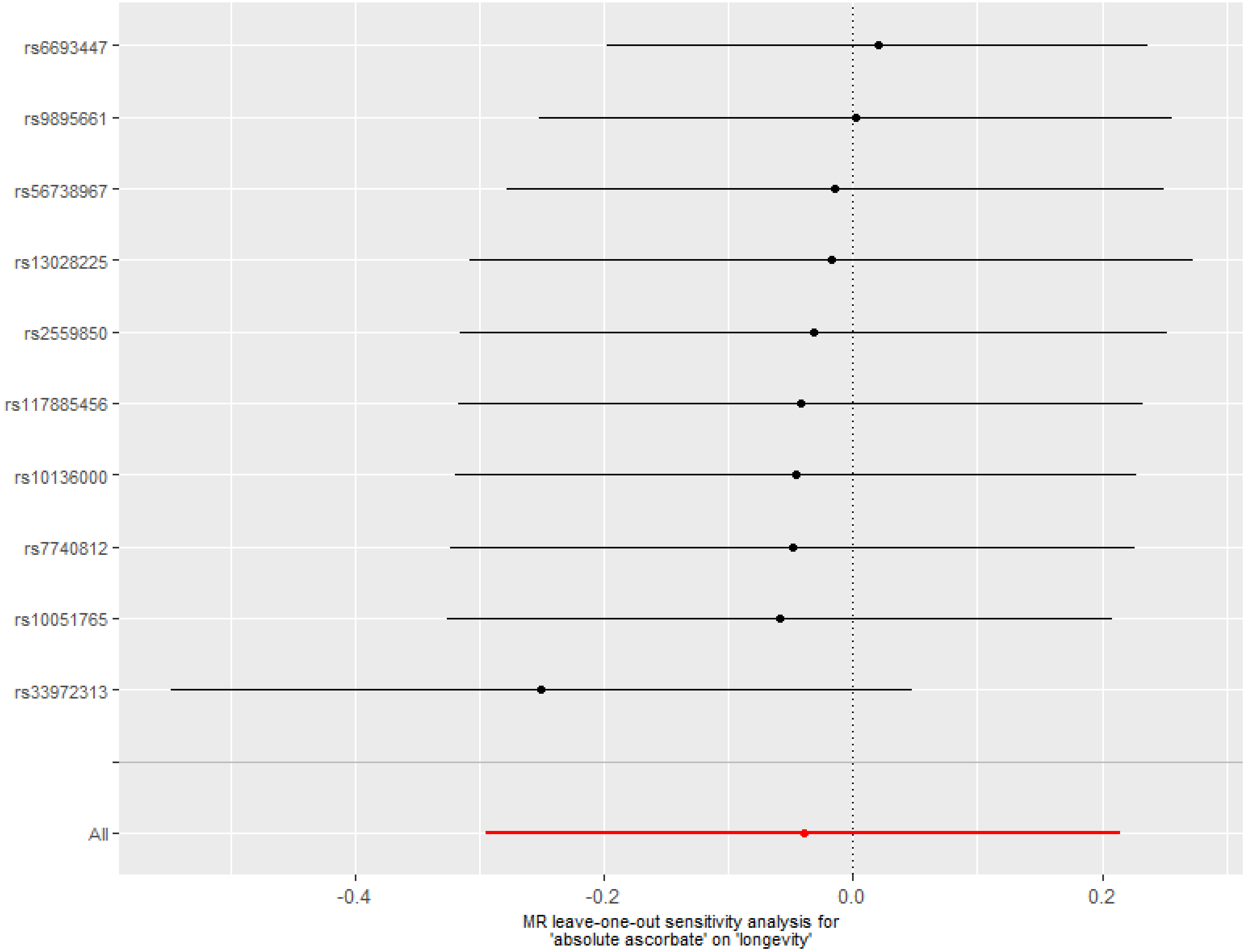

Online Supporting Material

Supplemental Figure S3: Leave-one-out permutations for the association of absolute circulating lycopene with longevity.

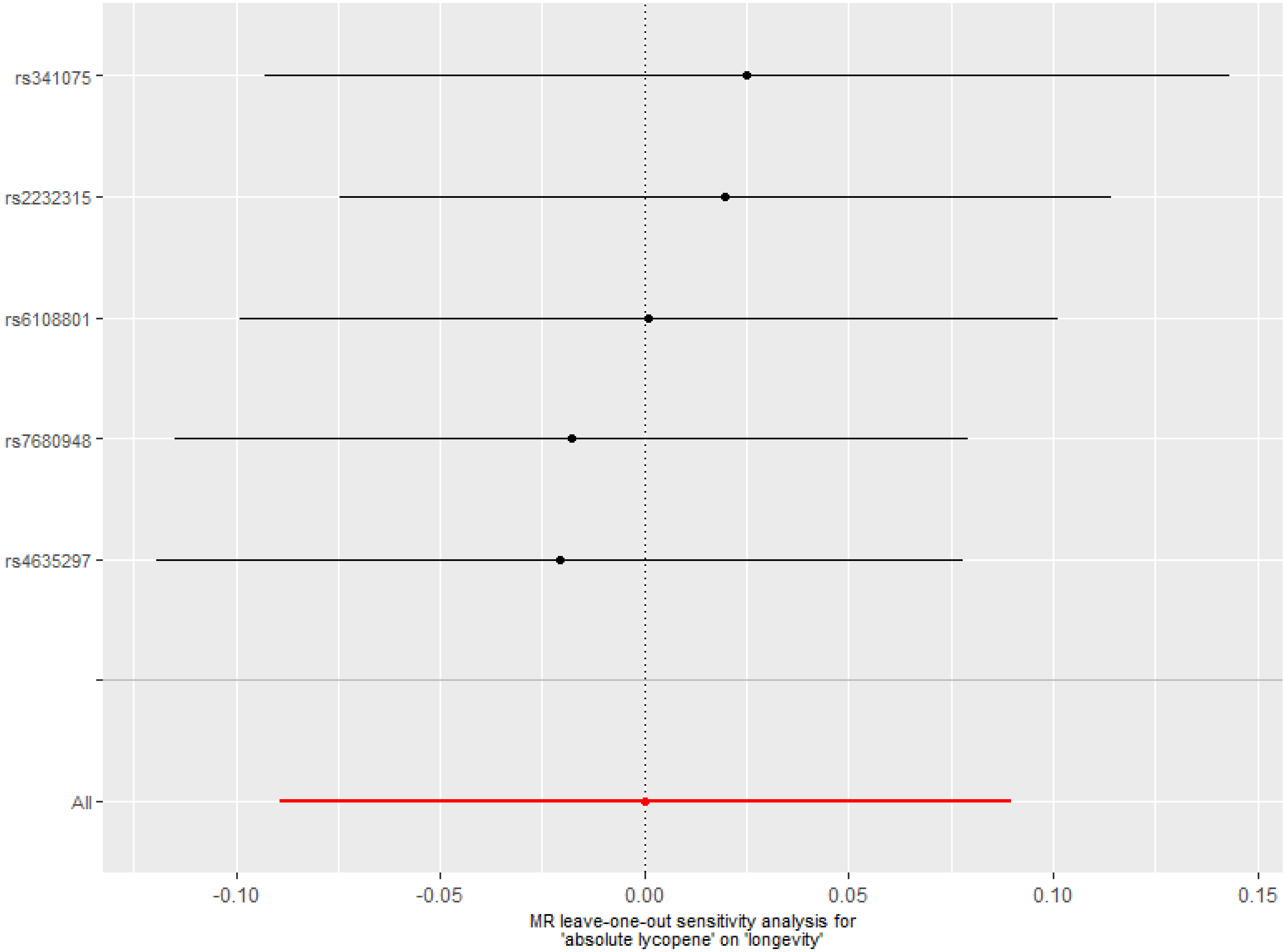

Online Supporting Material

Supplemental Figure S4: Leave-one-out permutations for the association of absolute circulating selenium with longevity.

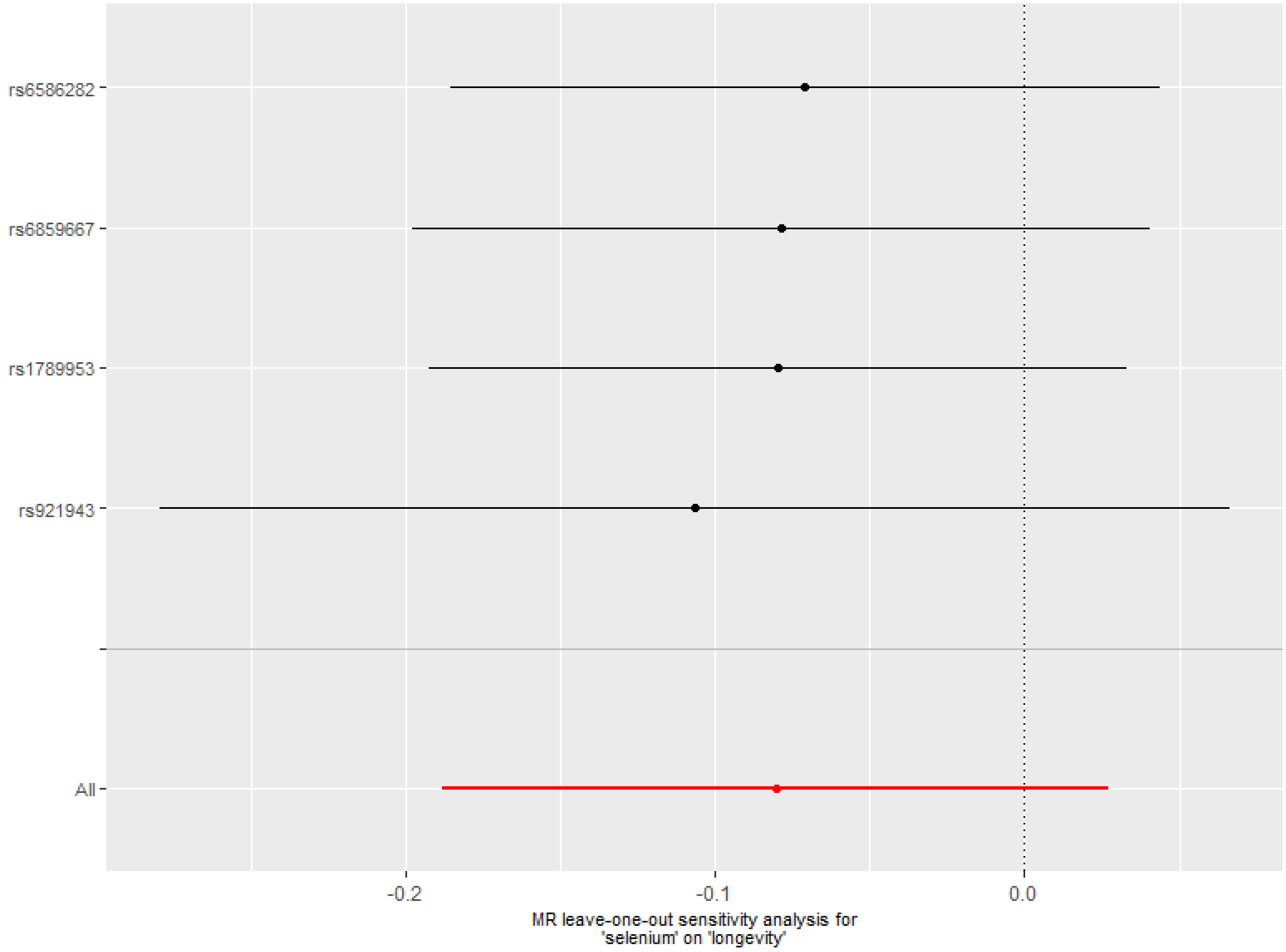

Online Supporting Material

Supplemental Figure S5: Leave-one-out permutations for the association of absolute circulating beta-carotene with longevity.

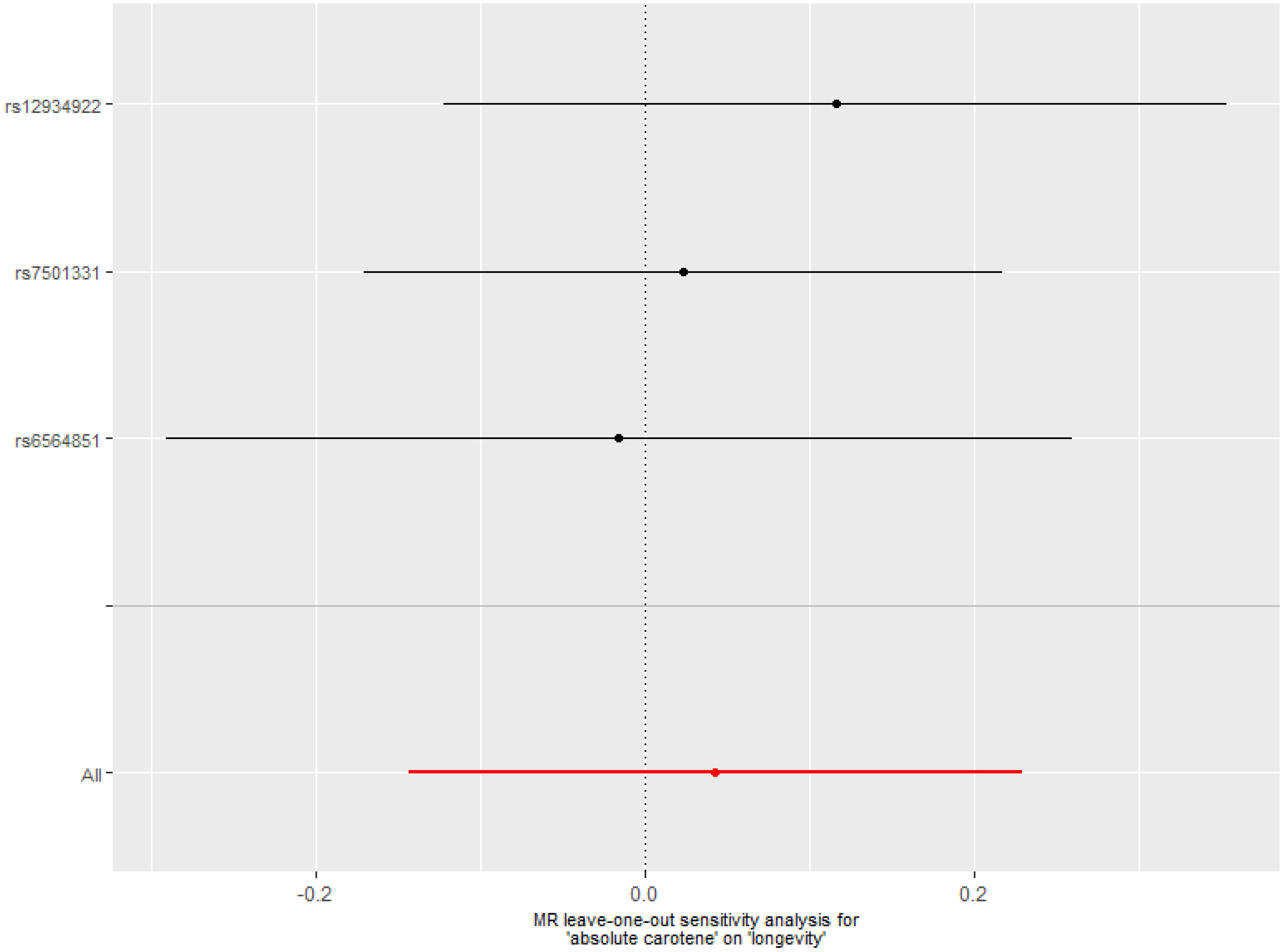

Online Supporting Material

Supplemental Figure S6: Leave-one-out permutations for the association of alpha-tocopherol metabolite with longevity.

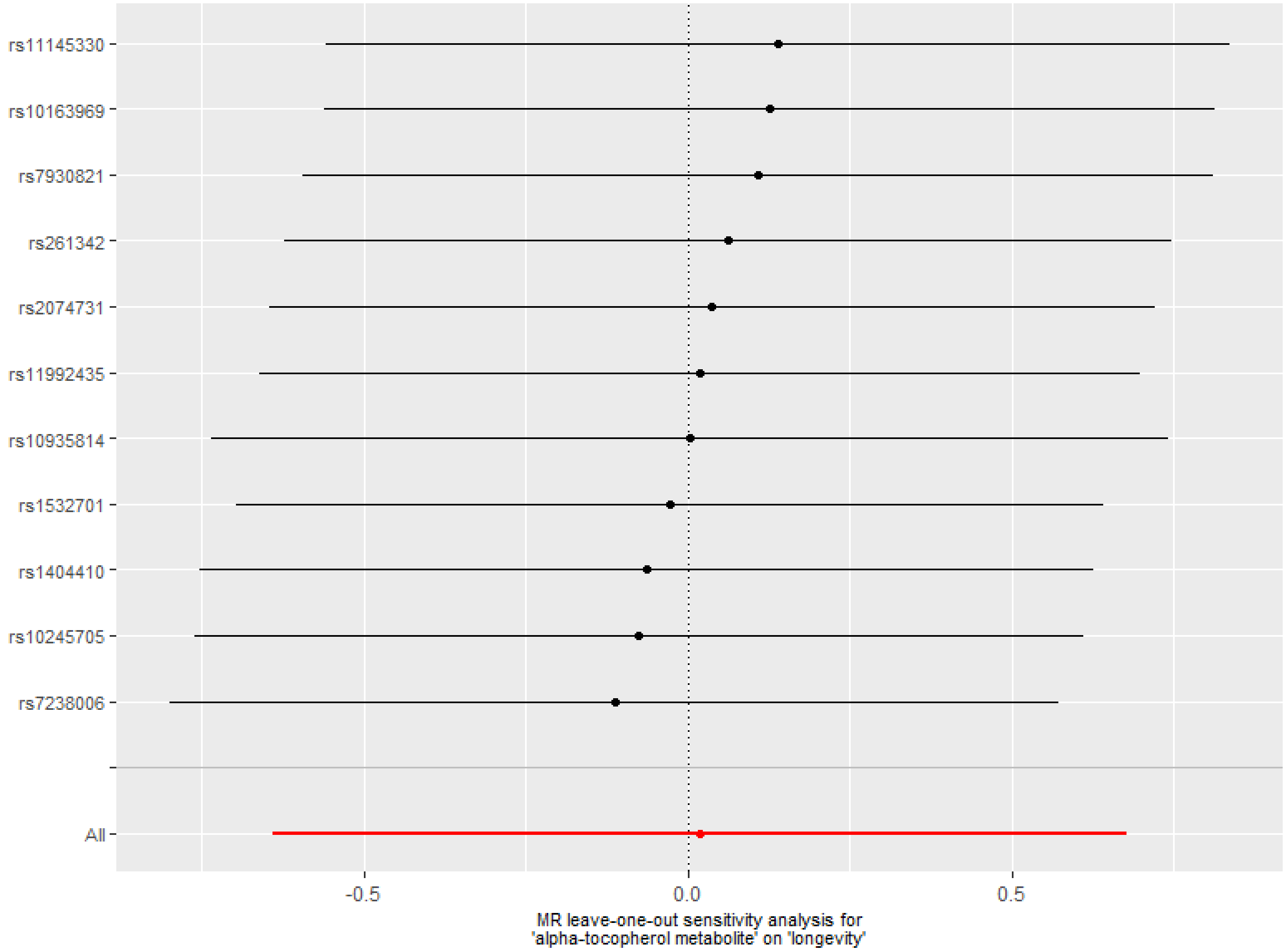

Online Supporting Material

Supplemental Figure S7: Leave-one-out permutations for the association of gamma-tocopherol metabolite with longevity.

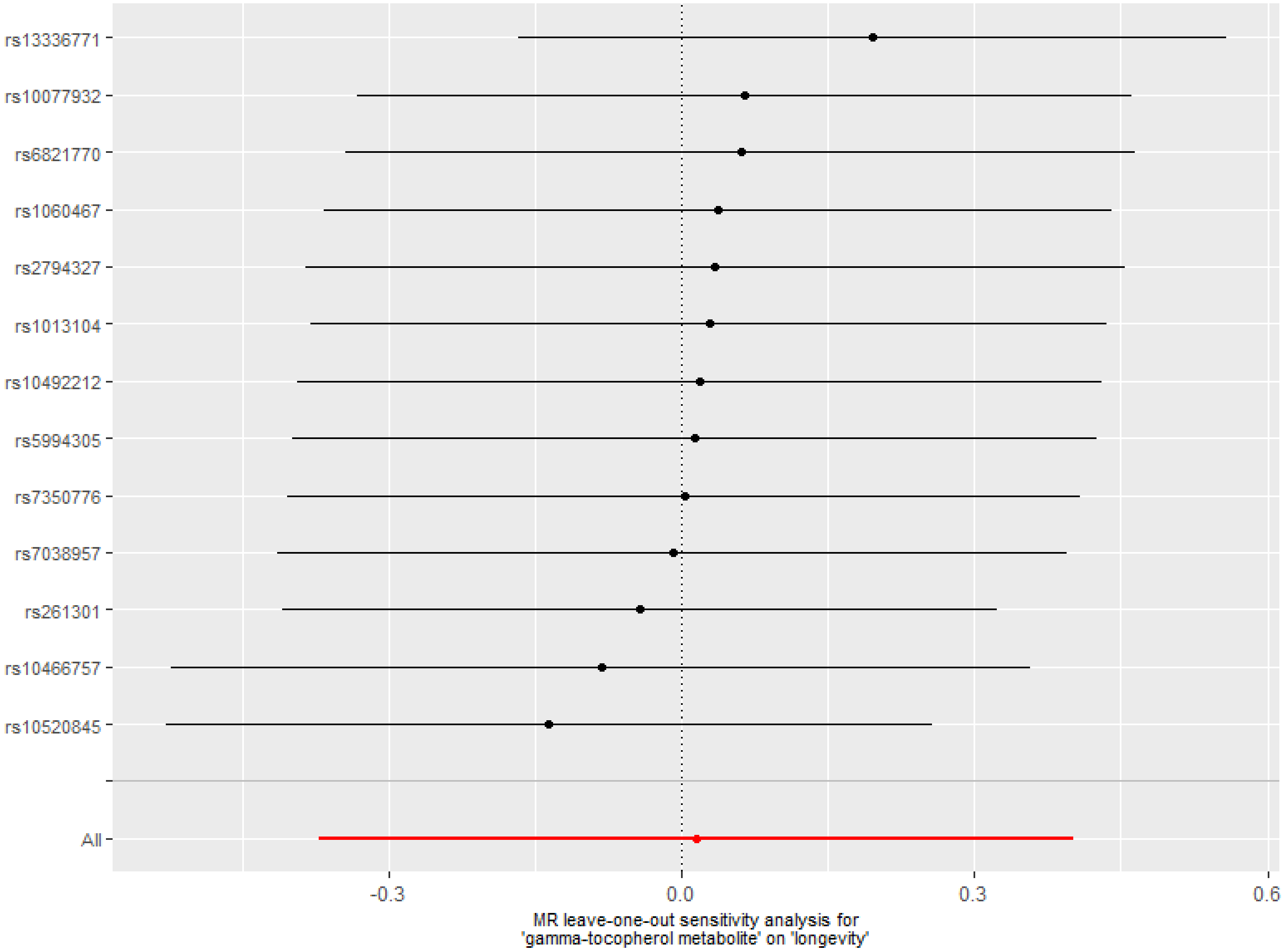

Online Supporting Material

Supplemental Figure S8: Leave-one-out permutations for the association of circulating retinol metabolite with longevity.

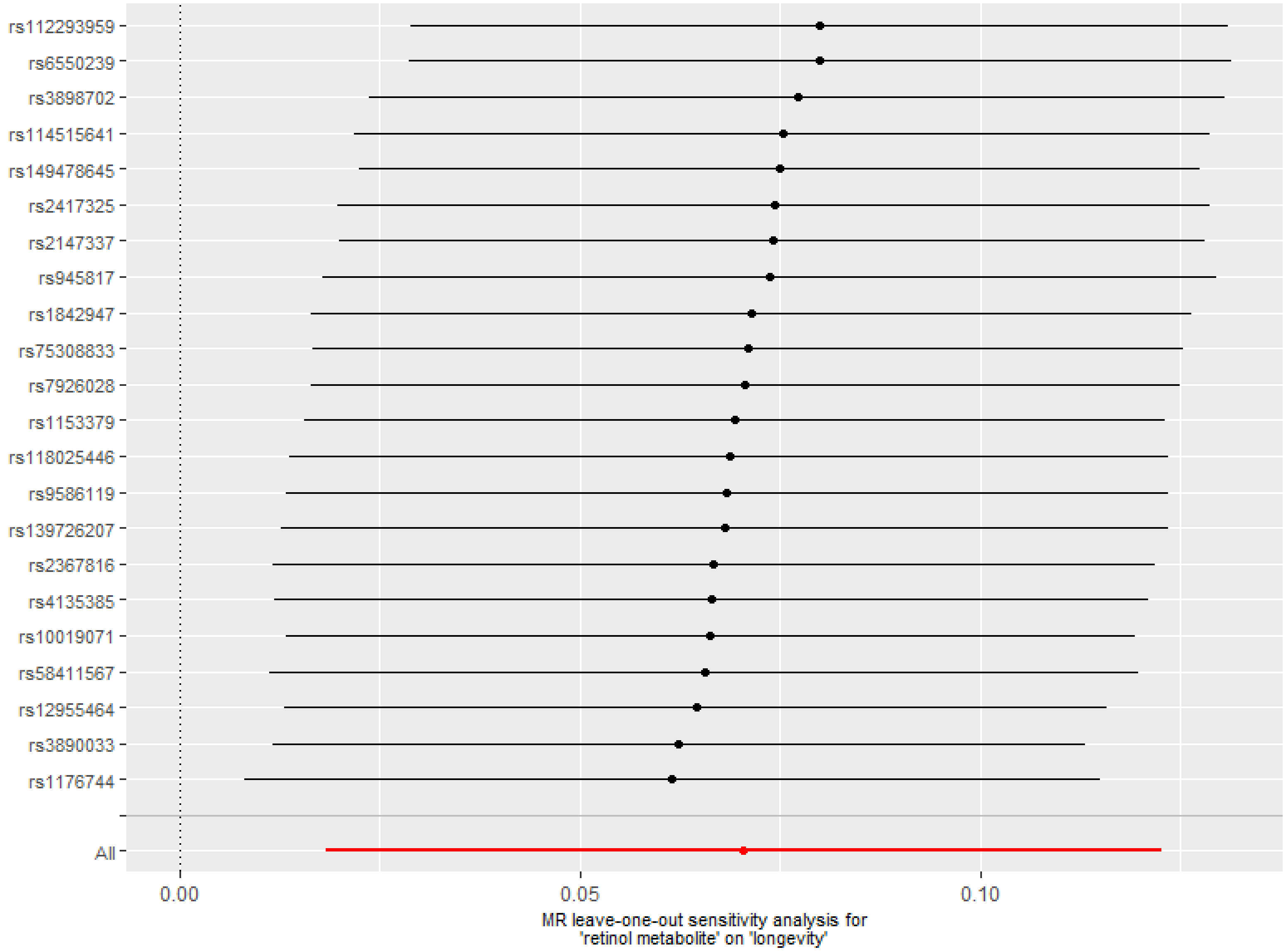

Online Supporting Material

Supplemental Figure S9: Leave-one-out permutations for the association of circulating ascorbate metabolite with longevity.

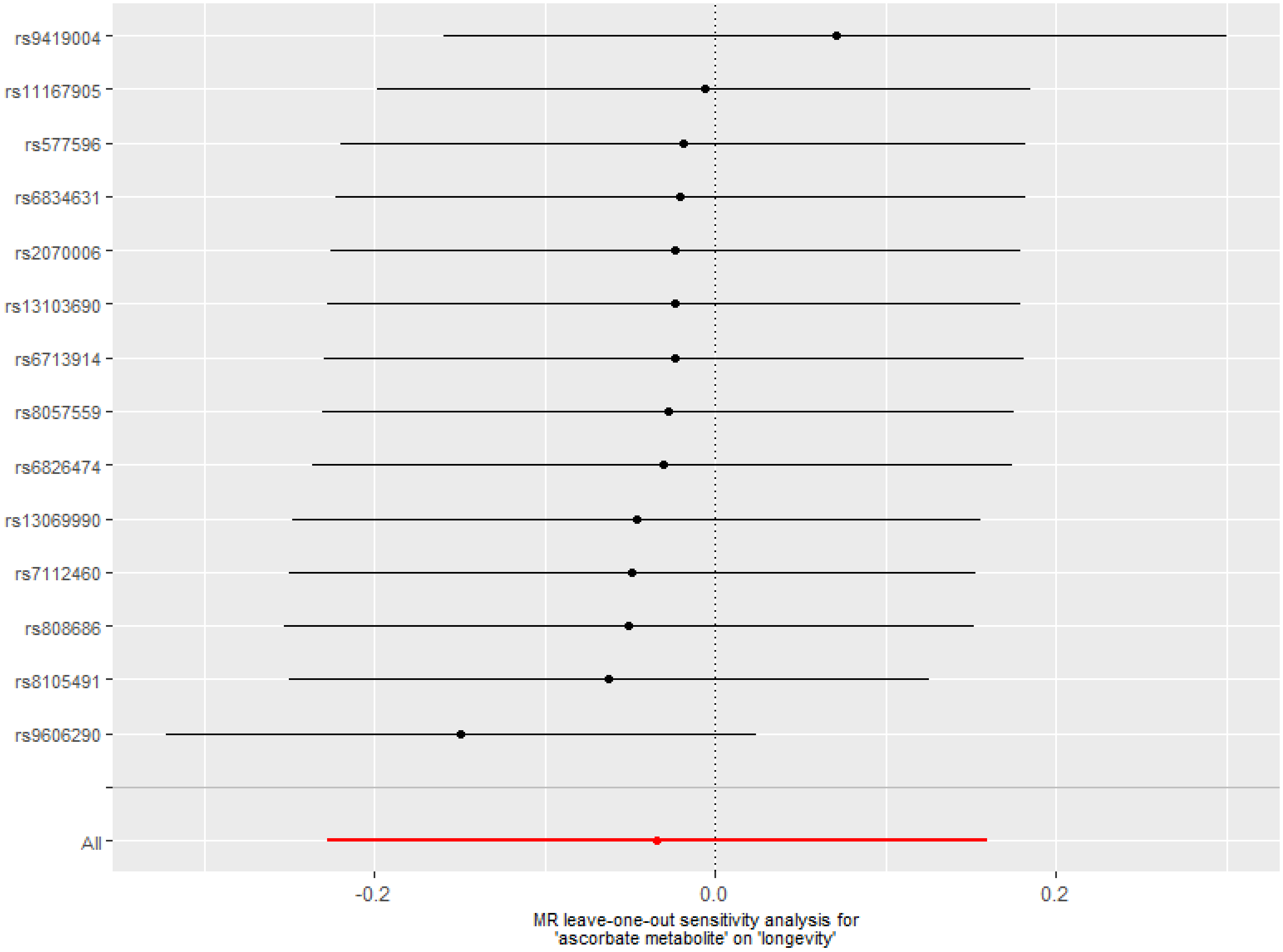

Supplement: Supplementary Materials — Supplemental Table S1: post hoc power calculation for the main IVW analyses on circulating antioxidants and longevity. Supplemental Table S2: cohort characteristics and exclusion criteria for the individual longevity GWAS. Supplemental Table S3: cohort characteristics and exclusion criteria for the parental lifespan GWAS. Supplemental Table S4: effect estimates for the associations of the included instrumental variants with absolute circulating antioxidants and longevity outcome. Supplemental Table S5: effect estimates for the associations of the selected instrumental variants with circulating antioxidant metabolites and longevity outcome. Supplemental Table S6: IVW findings and sensitivity analyses for genetically predicted per unit increase in circulating antioxidants and parental lifespan outcome. Supplemental Table S7: details of GWAS datasets used to test associations of antioxidant-instrumenting SNPs with potential confounders and other determinants of longevity. Supplemental Table S8: comparison of the impacts on circulating antioxidant levels between genetic instruments and antioxidant supplementation. Supplemental Figure S1: three principal assumptions in MR study. Supplemental Figure S2: leave-one-out permutations for the association of absolute circulating ascorbate with longevity. Supplemental Figure S3: leave-one-out permutations for the association of absolute circulating lycopene with longevity. Supplemental Figure S4: leave-one-out permutations for the association of absolute circulating selenium with longevity. Supplemental Figure S5: leave-one-out permutations for the association of absolute circulating beta-carotene with longevity. Supplemental Figure S6: leave-one-out permutations for the association of alpha-tocopherol metabolite with longevity. Supplemental Figure S7: leave-one-out permutations for the association of gamma-tocopherol metabolite with longevity. Supplemental Figure S8: leave-one-out permutations for the association of circulating [file 4012603.f1.zip › Supplemental Figures S1-9.pdf]
