# Supplementary material for: Association between Circulating Antioxidants and Longevity: Insight from Mendelian Randomization Study
Source: Biomed Res Int. 2022 Jan 29;2022:4012603. doi: 10.1155/2022/4012603 (PMC8817834; doi:10.1155/2022/4012603)
Supplement: Supplementary Materials — Supplemental Table S1: post hoc power calculation for the main IVW analyses on circulating antioxidants and longevity. Supplemental Table S2: cohort characteristics and exclusion criteria for the individual longevity GWAS. Supplemental Table S3: cohort characteristics and exclusion criteria for the parental lifespan GWAS. Supplemental Table S4: effect estimates for the associations of the included instrumental variants with absolute circulating antioxidants and longevity outcome. Supplemental Table S5: effect estimates for the associations of the selected instrumental variants with circulating antioxidant metabolites and longevity outcome. Supplemental Table S6: IVW findings and sensitivity analyses for genetically predicted per unit increase in circulating antioxidants and parental lifespan outcome. Supplemental Table S7: details of GWAS datasets used to test associations of antioxidant-instrumenting SNPs with potential confounders and other determinants of longevity. Supplemental Table S8: comparison of the impacts on circulating antioxidant levels between genetic instruments and antioxidant supplementation. Supplemental Figure S1: three principal assumptions in MR study. Supplemental Figure S2: leave-one-out permutations for the association of absolute circulating ascorbate with longevity. Supplemental Figure S3: leave-one-out permutations for the association of absolute circulating lycopene with longevity. Supplemental Figure S4: leave-one-out permutations for the association of absolute circulating selenium with longevity. Supplemental Figure S5: leave-one-out permutations for the association of absolute circulating beta-carotene with longevity. Supplemental Figure S6: leave-one-out permutations for the association of alpha-tocopherol metabolite with longevity. Supplemental Figure S7: leave-one-out permutations for the association of gamma-tocopherol metabolite with longevity. Supplemental Figure S8: leave-one-out permutations for the association of circulating [file 4012603.f1.zip › Supplemental Tables S1-8.pdf]

Online Supporting Material

Association Between Circulating Antioxidants and Longevity: Insight from Mendelian Randomization Study

Zhimin Yu, Fangfang Zhang, Chengkai Xu, Yanggan Wang

Online Supplement

Online Supporting Material

Supplemental Table S1: Post-hoc power calculation for the main IVW analyses on dietary-derived circulating antioxidants and longevity.

Antioxidant	N*	R^2 of exposure by genetic instruments†	Effect estimate (Beta according to IVW)	Significance level	Power§	PMID
**Absolute circulating antioxidants**						
Ascorbate	1,012,240	0.018	-0.05	0.05	100%	33203707
Lycopene	1,012,240	0.301	0.07	0.05	100%	26861389
β-carotene	1,012,240	0.090	-0.24	0.05	100%	25343990
Retinol	1,012,240	0.023	0.30	0.05	100%	23134893
Selenium	1,012,240	0.059	0.00	0.05	11%	21878437
**Circulating antioxidants metabolites**						
Ascorbate	1,012,240	0.186	-0.03	0.05	100%	24816252
α-tocopherol	1,012,240	0.033	0.08	0.05	100%	24816252
γ-tocopherol	1,012,240	0.150	-0.62	0.05	100%	24816252
Retinol	1,012,240	0.048	0.17	0.05	100%	28263315

* Sample size and ratio of cases to controls according to the parental lifespan GWAS.

† The proportion of phenotypic variance explained by genetic instruments as reported in GWASs or calculated on the different circulating antioxidants.

§ Power value was calculated using an online power calculation tool (https://sb452.shinyapps.io/power/).

Abbreviations: IVW, inverse-variance weighted.

Online Supporting Material

Supplemental Table S2: Cohort characteristics and exclusion criteria for the individual longevity GWAS (*90th vs. 60th percentile age dataset*).

Cohort	PMID reference	Ancestry	Study design	Cases 90th percentile age			All controls			Exclusion criteria
				N	Mean age (SD, years)	Male (%)	N	Mean age (SD, years)	Male (%)	
100-plus Study	30362018	European	Population-based	293	101.42 (1.69)	27.3	NA	NA	NA	1) Ethnic outliers;<br>2) Duplicates;<br>3) Related individuals
Amsterdam dementia cohort	29562540	European	Population-based	5	95.49 (2.67)	80.0	898	60.07 (8.83)	60.65	1) Ethnic outliers;<br>2) Duplicates;<br>3) Related individuals
Age/Gene Environment Susceptibility Study	17351290	European	Population-based	300	95.03 (2.45)	59.0	1001	81.32 (3.30)	12.30	1) Gender mismatch;<br>2) Mismatch previous genotypes
CEPH centenarian cohort (controls from Supplementation in Vitamins and Mineral Antioxidants study)	11280044	European	Population-based	1234	102.35 (3.03)	18.6	831	56.1 (6.2)	40.80	1) Ethnic outliers;<br>2) Heterozygosity;<br>3) Gender mismatches;<br>4) Duplicates;<br>5) Related individuals
Cardiovascular Health Study	1669507	European	Population-based	905	94.0 (3.28)	61.1	558	77.76 (3.93)	14.20	1) Gender mismatches
Danish longevity study (controls from Genomics of extremely Overweight Young Adults study)	28449061<br>12184889<br>21935397	European	Population-based	960	100.17 (2.90)	25.4	1917	26.52 (5.54)	27.96	1) Ethnic outliers;<br>2) Heterozygosity;<br>3) Gender mismatches;<br>4) Duplicates;<br>5) Related individuals;<br>6) Mismatch previous genotypes
Framingham Heart Study	17372189	European	Population-based	332	94.44 (3.42)	55.1	1444	75.95 (5.11)	39.63	1) Ethnic outliers;<br>2) Heterozygosity;<br>3) Mendelian errors
GEnetics of Healthy Aging (Danish subsample) (controls from Genomics of extremely Overweight Young Adults study)	21871552	European	Family-based	451	97.76 (2.88)	32.2	900	26.56 (5.54)	28.89	1) Ethnic outliers;<br>2) Heterozygosity;<br>3) Gender mismatches;<br>4) Duplicates;<br>5) Related individuals;<br>6) Mismatch previous genotypes
GEnetics of Healthy Aging (Dutch subsample)	21871552	European	Family-based	182	98.77 (2.97)	35.7	NA	NA	NA	1) Gender mismatches
GEnetics of Healthy Aging	21871552	European	Family-based	271	98.88 (3.03)	31.0	358	63.4 (6.8)	35.00	1) Gender mismatches

Online Supporting Material

(French subsample)										
GEnetics of Healthy Aging (Italian subsample)	21871552	European	Family-based	182	98.96 (2.68)	27.5	184	62.6 (6.0)	27.70	1) Gender mismatches
Health and Retirement Study	24671021	European	Population-based	361	93.8 (3.0)	59.6	3312	77.3 (3.7)	34.50	1) Ethnic outliers;<br>2) Heterozygosity;<br>3) Gender mismatches;<br>4) Duplicates;<br>5) Related individuals
Longitudinal aging study of Amsterdam	21216744	European	Population-based	75	94.16 (3.67)	57.3	1373	69.85 (6.97)	45.96	1) Ethnic outliers;<br>2) Duplicates;<br>3) Related individuals
Long Life Family Study	21258136	European	Family-based	1110	97.7 (3.81)	53.0	552	77.4 (3.14)	38.20	1) Ethnic outliers;<br>2) Heterozygosity;<br>3) Gender mismatches
Leiden Longevity Study	16251894<br>19682117	European	Family-based	855	97.73 (3.47)	40.7	712	67.62 (7.03)	40.70	1) Gender mismatches;<br>2) Family mismatches
Longevity Gene Project	16602826<br>24522433	European	Population-based	548	96.3 (4.7)	32.0	584	68.2 (5.8)	35.50	1) Gender mismatches;<br>2) Family mismatches
Osteoporotic Fractures in Men Study	16085466<br>16084776	European	Population-based	1171	93.0 (2.8)	100.0	386	75.1 (2.9)	100.00	1) Ethnic outliers;<br>2) Gender mismatches;<br>3) Related individuals
Newcastle 85+ Study (controls from Wellcome Trust Case Control Consortium 2 study)	20028777	European	Population-based	215	93.28 (1.36)	62.3	5159	20.07 (22.47)	50.53	1) Ethnic outliers;<br>2) Heterozygosity;<br>3) Gender mismatches;<br>4) Duplicates;<br>5) Related individuals
Rotterdam study	21877163	European	Population-based	774	94.96 (3.34)	42.9	2965	78.22 (5.31)	33.22	1) Ethnic outliers;<br>2) Heterozygosity;<br>3) Gender mismatches;<br>4) Duplicates;<br>5) Related individuals
Study of Osteoporotic Fracture	2404146	European	Population-based	812	96.44 (2.17)	0.0	354	80.67 (2.24)	0.00	1) Ethnic outliers;<br>2) Gender mismatches;<br>3) Related individuals
Vitality 90+ project (controls from Cardiovascular Risk in Young Finns Study)	16137286	European	Population-based	226	92.93 (1.13)	26.1	1995	41.89 (4.98)	44.30	1) Heterozygosity;<br>2) Gender mismatches;<br>3) Related individuals

Online Supporting Material

Supplemental Table S3: Cohort characteristics and exclusion criteria for the parental lifespan GWAS.

Cohort	PMID reference	Ancestry	Study design	N	No. of Father	No. of Mother	Mean age (years)	Exclusion criteria
**UK Biobank**	25826379	European	Population-based	691,621	339,732	351,889	74.8	Adopted or unclear about adoption status; not report parental ages; both parents died before age of 40; one of each pair from related individuals (third degree or closer)
**LifeGen cohort**								
Age and Cognitive Performance Research Centre (ACPRC)	DOI: 10.1080/ 13825580490511116	European	Population-based	2,873	1,421	1,452	73.5	Ethnic outliers; duplicates; Gender mismatch; IBS incompatible with pedigree
Atherosclerosis Risk in Communities Study (ARIC_EA)	2646917	European	Population-based	16,890	8,342	8,548	73.0	-
Austrian Stroke Prevention Family Study (ASPS-Fam)	25443291, 25309438	European	Population-based	295	144	151	76.1	Ethnic outliers; duplicates; Gender mismatch; cryptic relatedness
BASE-II (BASE-II)	promised	-	-	2,611	1,163	1,448	77.2	-
Cardiovascular Health Study (CHS_EA)	1669507	European	Population-based	5,609	2,818	2,791	75.2	Baseline CHD, CHF, peripheral vascular disease, valvular heart disease, stroke, or TIA; non-EA; discordant with known sex or prior genotyping, lack of consent
Cilento Study (CILENTO)	17476112 19550436	European	Cross-sectional population based	1,247	625	622	77.2	Ethnic outliers; gender mismatch; IBS incompatible with pedigree
Cohorte Lausannois (CoLaus)	18366642	European	Population-based	10,104	5,051	5,053	73.8	none
Estonian Genome Centre University of Tartu (EGCUT ExomeCore)	24518929	European	Population-based	7,719	3,692	4,027	60.8	Ethnic outliers; duplicates; Gender mismatch; IBS incompatible with pedigree
Estonian Genome Centre University of Tartu (EGCUT omni)	19424496	European	Population-based	10,530	5,030	5,500	67.5	Ethnic outliers; duplicates; gender mismatch; IBS incompatible with pedigree
Erasmus Rucphen Family Study (ERF)	15845033	European	Family-based	1,931	956	975	71.5	Ethnic outliers; duplicates; gender mismatch; IBS incompatible with pedigree
Genetic Study of Atherosclerosis Risk (GeneSTAR_EA)	17950799	European American	Family-based	2,073	1,028	1,045	69.8	ethnic outliers; gender mismatch; >5% Mendelian inconsistencies

Online Supporting Material

Generation Scotland: Scottish Family Health Study (GS:SFHS)	22786799 (GS10K)	European	Family-based	-	-	-	-	Ethnic/PCs Outliers; duplicates
ipsych (iPSYCH)	-	European	Population based	119,265	61,224	58,041	51.4	Duplicates, gender mismatch
Micro-isolates in South Tyrol (MICROS)	-	European	Population-based	1,601	782	819	70.1	Ethnic outliers; duplicates; gender mismatch; IBS incompatible with pedigree
MRC National Survey of Health and Development (NSHD)	16204333, 21345808 , 23977022	European	Prospective birth cohort	4,424	2,199	2,225	74.1	Sex mismatch
Nijmegen Biomedical Study (NBS)	28082374	European	Population based	8,695	4,352	4,343	74.1	Ethnic outliers; duplicates; gender mismatch; relatedness PI_HAT>0.25
Netherlands Twin Registry (NTR)	-	European	Twin Families - Population-based	8,892	4372	4520	68.4	Ethnic outliers, gender mismatch, IBS incompatible with pedigree, duplicates, >5sd Mendelian errors, CQC < 0.40, callrate > 0.90
Ogliastra Genetic Park (OGP)	20004756	European	Population-based	1,266	636	630	72.7	Ethnic outliers; duplicates; gender mismatch; IBS incompatible with pedigree
Orkney Complex Disease Study (ORCADES HAP300)	18760389	European	Population-based	996	488	508	72.7	Ethnic outliers; duplicates; gender mismatch; IBS incompatible with pedigree
Prospective Study of Pravastatin in the Elderly at Risk (PROSPER)	10569329; 12457784; 21977987	European	RCT/Population-based	9,832	4915	4917	74.1	Ethnic outliers; duplicates; gender mismatch; IBS incompatible with pedigree
Queensland Institute of Medical Research (QIMR Berghofer)	-	European	Population-based (largely twin families from Australian Twin Registry)	8,774	4367	4407	69.8	Ethnic outliers; gender mismatch; IBD checks against expected and deduced pedigrees
Rotterdam Study (RS1)	26386597	European	Population-based	10,745	5,342	5403	75.4	Ethnic outliers; duplicates; gender mismatch
Rotterdam Study (RS2)	26386597	European	Population-based	3,373	1659	1714	76.3	Ethnic outliers; duplicates; gender mismatch
Shetland Complex Disease Study (VIKING)	-	European	Population-based	1,226	610	616	71.6	Outliers; duplicates
Vitality90 (Vitality90)	16137286 26436701	European	Population-based, nonagenarians	444	215	229	71.8	Gender mismatch (n=1), Cryptic relatedness (n=0), MDS outliers removed (n=13)

Online Supporting Material

Women's Genome Health Study (WGHS)	18070814?	European	Population based	35,401	17308	18093	77.8	Multidimensional scaling in PLINK using 1443 ancestry informative markers to verify self-reported European ancestry.

Supplemental Table S4: Effect estimates for the associations of the selected instrumental variants with absolute circulating antioxidants and longevity across two datasets.

SNP	Antioxidant	Chr	Pos	Nearest Gene	EA	OA	EAF	F statistic	Exposure			Longevity outcomes					
												90th vs. 60th percentile age			Parental lifespan		
									Beta	SE	P-value	Beta	SE	P-value	Beta	SE	P-value
rs33972313	ascorbate	5	138715502	*SLC23A1*	C	T	0.960	122.10	0.360	0.018	4.61E-90	0.075	0.057	0.19	0.003	0.0107	0.78
rs10051765	ascorbate	5	176799992	*RGS14*	C	T	0.342	10.33	0.039	0.007	3.64E-09	0.017	0.0221	0.43	0.002	0.004	0.71
rs10136000	ascorbate	14	105253581	*AKT1*	A	G	0.283	10.80	0.040	0.007	1.33E-08	0.006	0.025	0.80	-0.002	0.004	0.62
rs117885456	ascorbate	12	96249111	*SNRPF*	A	G	0.087	14.59	0.078	0.012	1.70E-11	9.00E-04	0.045	0.98	-0.013	0.007	0.07
rs13028225	ascorbate	2	220031255	*SLC23A3*	T	C	0.857	38.57	0.102	0.009	2.38E-30	-0.016	0.029	0.55	0.006	0.006	0.31
rs2559850	ascorbate	12	102093459	*CHPT1*	A	G	0.598	24.44	0.058	0.006	6.30E-20	-0.007	0.020	0.73	0.001	0.004	0.79
rs56738967	ascorbate	16	79740541	*MAF*	C	G	0.321	11.06	0.041	0.007	7.62E-10	-0.023	0.021	0.25	0.003	0.004	0.42
rs6693447	ascorbate	1	2330190	*RER1*	T	G	0.551	11.36	0.039	0.006	6.25E-10	-0.050	0.020	0.01	-0.009	0.004	0.02
rs7740812	ascorbate	6	52725787	*GSTA5*	G	A	0.594	10.51	0.038	0.006	1.88E-09	0.005	0.020	0.77	-0.001	0.004	0.79
rs12934922	β-carotene	16	81301694	*BCMO1*	T	A	0.44	60.21	0.139	0.020	5.90E-10	-0.010	0.021	0.63	0.002	0.004	0.60
rs6564851	β-carotene	16	81264597	*BCMO1*	G	T	0.36	98.67	0.149	0.020	1.60E-24	0.014	0.019	0.48	0.006	0.004	0.15
rs7501331	β-carotene	16	81314496	*BCMO1*	T	C	0.24	17.69	-0.067	0.020	1.60E-05	-0.019	0.023	0.42	-0.005	0.005	0.29
rs2232315	lycopene	2	169757432	*G6PC2*	A	G	0.03	25.01	0.740	0.150	1.26E-06	-0.126	0.105	0.23	-0.015	0.015	0.34
rs341075	lycopene	11	72257963	–	A	G	0.02	24.91	-0.870	0.170	5.75E-07	0.029	0.061	0.63	-0.014	0.012	0.22
rs4635297	lycopene	15	38327408	*BC039545*	A	C	0.08	25.97	0.260	0.050	6.46E-07	0.025	0.028	0.37	-0.005	0.005	0.30
rs6108801	lycopene	20	10989519	–	C	T	0.04	27.32	-0.480	0.090	4.07E-07	0.002	0.058	0.97	-0.004	0.011	0.70

Online Supporting Material

rs7680948	lycopene	4	140447105	*SETD7*	A	C	0.20	38.52	-0.190	0.030	4.97E-09	-0.020	0.022	0.39	-0.009	0.004	0.03
rs10882272	retinol	10	95348182	*RBP4*	C	T	0.35	34.57	-0.030	0.010	7.00E-15	0.006	0.020	0.77	-0.003	0.004	0.50
rs1667255	retinol	18	29187279	*TTR*	C	A	0.31	34.57	0.030	0.010	6.00E-14	-0.037	0.020	0.06	-0.003	0.004	0.51
rs1789953	selenium	21	44482936	*CBS, PKNOX1, U2AF1, WDR4*	T	C	0.14	30.47	0.162	0.029	3.40E-08	-0.014	0.030	0.64	0.005	0.006	0.36
rs6586282	selenium	21	44478497	*CBS, PKNOX1, U2AF1, STAG3OS*	T	C	0.17	34.69	-0.160	0.027	3.96E-09	0.024	0.026	0.35	0.011	0.005	0.02
rs6859667	selenium	5	78745042	*HOMER1, JMY*	T	C	0.96	47.89	-0.360	0.052	4.40E-12	0.032	0.046	0.50	0.010	0.010	0.33
rs921943	selenium	5	78316476	*DMGDH, ARSB, BHMT2*	T	C	0.29	172.66	0.295	0.022	1.90E-39	-0.019	0.021	0.36	-0.007	0.004	0.13

Abbreviations: Chr, Chromosome; EA, effect allele; EAF, effect allele frequency; OA, other allele; NA, not available; Pos, position; SE, standard error; SNP, single nucleotide polymorphism.

The unit of beta coefficients is log-odds per allele.

Online Supporting Material

Supplemental Table S5: Effect estimates for the associations of the selected instrumental variants with circulating antioxidant metabolites and longevity across two datasets.

SNP	Antioxidant	Chr	Pos	Nearest Gene	EA	OA	EAF	F statistic	Exposure			Longevity outcomes					
												90th vs. 60th percentile age			Parental lifespan		
									Beta	SE	P-value	Beta	SE	P-value	Beta	SE	P-value
rs10163969	alpha-tocopherol	18	9067439	*NDUFV2*	T	G	0.04	19.36	-0.040	0.010	9.39E-06	0.048	0.047	0.31	NA	NA	NA
rs10245705	alpha-tocopherol	7	26129672	*AC004520.1*	T	C	0.02	27.25	-0.070	0.010	1.95E-07	-0.081	0.085	0.34	-0.005	0.015	0.73
rs10935814	alpha-tocopherol	3	150352873	*SELENOT*	A	G	0.10	19.87	-0.040	0.010	9.44E-06	-0.003	0.030	0.91	-0.004	0.006	0.53
rs11145330	alpha-tocopherol	9	71733603	*TJP2*	A	C	0.89	22.70	0.030	0.010	1.95E-06	-0.029	0.031	0.34	0.003	0.006	0.57
rs11992435	alpha-tocopherol	8	81526083	*RN7SL107P*	A	G	0.95	20.63	0.030	0.010	6.38E-06	0.001	0.040	0.99	-0.005	0.008	0.57
rs1404410	alpha-tocopherol	7	124248950	*RNU6-102P*	C	G	0.79	20.60	-0.020	0.010	4.57E-06	-0.018	0.023	0.44	-0.005	0.005	0.27
rs1532701	alpha-tocopherol	16	55698027	*SLC6A2*	A	G	0.55	20.85	0.010	0.000	5.07E-06	0.015	0.020	0.43	0.005	0.004	0.23
rs2074731	alpha-tocopherol	22	30733914	*SF3A1*	A	C	0.17	22.26	-0.020	0.000	2.31E-06	0.004	0.025	0.86	0.005	0.005	0.34
rs261342	alpha-tocopherol	15	58731153	*LIPC*	C	G	0.79	20.86	-0.020	0.000	5.41E-06	0.010	0.025	0.67	0.004	0.005	0.38
rs7238006	alpha-tocopherol	18	31927898	*RP11-379L18.3*	T	C	0.93	24.30	0.030	0.010	6.77E-07	0.047	0.036	0.19	0.005	0.007	0.54
rs7930821	alpha-tocopherol	11	19142695	*ZDHHC13*	T	C	0.02	19.98	0.070	0.010	7.53E-06	-0.044	0.067	0.52	-0.021	0.014	0.13
rs10077932	gamma-tocopherol	5	2450679	*LINC02142*	T	C	0.14	21.35	-0.040	0.010	4.08E-06	0.032	0.029	0.26	0.013	0.005	0.01
rs1013104	gamma-tocopherol	17	12187236	*RP11-471L13.2*	T	C	0.44	21.16	-0.020	0.000	3.83E-06	0.008	0.019	0.69	0.008	0.004	0.03
rs10466757	gamma-tocopherol	12	24984990	*BCAT1*	A	T	0.16	19.66	0.060	0.010	9.56E-06	0.022	0.022	0.32	NA	NA	NA
rs10492212	gamma-tocopherol	12	107296945	*EEF1B2P4*	T	C	0.16	19.53	-0.030	0.010	8.66E-06	0.002	0.025	0.94	0.002	0.005	0.77
rs10520845	gamma-tocopherol	5	16879933	*MYO10*	A	C	0.02	20.78	0.190	0.040	5.27E-06	0.135	0.077	0.08	-0.001	0.017	0.97
rs1060467	gamma-tocopherol	19	16024538	*CYP4F11*	A	G	0.59	26.81	0.020	0.000	2.61E-07	-0.014	0.020	0.48	-0.006	0.004	0.12
rs13336771	gamma-tocopherol	16	48318728	*LONP2*	A	C	0.17	20.15	0.060	0.010	7.39E-06	-0.059	0.026	0.03	0.007	0.005	0.17
rs261301	gamma-tocopherol	15	58686939	*ALDH1A2*	T	C	0.13	22.56	0.030	0.010	2.06E-06	0.050	0.028	0.07	0.003	0.006	0.63
rs2794327	gamma-tocopherol	1	3512341	*MEGF6*	T	C	0.67	19.69	-0.040	0.010	8.78E-06	0.008	0.024	0.74	0.000	0.004	0.99
rs5994305	gamma-tocopherol	22	30820707	*SEC14L2*	A	G	0.83	24.52	0.030	0.010	7.15E-07	0.002	0.025	0.95	-0.002	0.005	0.69
rs6821770	gamma-tocopherol	4	7732805	*SORCS2*	A	G	0.14	19.52	0.040	0.010	8.92E-06	-0.024	0.026	0.36	-0.006	0.005	0.25
rs7038957	gamma-tocopherol	9	106539439	*RNA5SP291*	T	C	0.83	21.43	-0.030	0.010	3.86E-06	-0.019	0.026	0.46	0.008	0.005	0.14
rs7350776	gamma-tocopherol	15	33689075	*RYR3*	C	G	0.70	21.12	0.020	0.010	3.86E-06	0.010	0.021	0.64	0.002	0.004	0.61
rs11167905	ascorbate	5	144906846	*PRELID2*	C	T	0.14	24.03	-0.080	0.020	9.83E-07	0.053	0.028	0.06	-0.001	0.005	0.87

Online Supporting Material

rs13069990	ascorbate	3	115620801	*LSAMP*	T	C	0.62	21.16	-0.050	0.010	4.44E-06	-0.017	0.020	0.40	-0.002	0.004	0.62
rs13103690	ascorbate	4	9972778	*SLC2A9*	G	T	0.47	20.77	0.050	0.010	5.20E-06	-0.016	0.019	0.42	-0.003	0.004	0.51
rs2070006	ascorbate	4	155513866	*FGA*	C	T	0.37	20.90	-0.050	0.010	4.76E-06	0.018	0.020	0.37	0.005	0.004	0.17
rs577596	ascorbate	22	25931606	*AL008721.1*	A	G	0.38	24.74	-0.060	0.010	6.68E-07	0.025	0.022	0.26	0.002	0.004	0.66
rs6713914	ascorbate	2	59523041	*AC007131.2*	C	T	0.44	26.13	-0.060	0.010	3.22E-07	0.013	0.019	0.49	0.000	0.004	0.98
rs6826474	ascorbate	4	96650120	*MANBA*	T	C	0.96	23.06	-0.140	0.030	1.56E-06	0.018	0.054	0.74	-0.004	0.012	0.71
rs6834631	ascorbate	4	122315665	*RP11-170N16.1*	G	T	0.04	23.93	-0.130	0.030	1.03E-06	0.048	0.048	0.31	-0.006	0.009	0.54
rs7112460	ascorbate	11	125751859	*HYLS1*	T	C	0.93	23.63	0.110	0.020	1.14E-06	0.037	0.040	0.36	0.011	0.008	0.17
rs8057559	ascorbate	16	73317172	*RP11-140I24.2*	T	C	0.96	19.74	0.140	0.030	9.10E-06	-0.043	0.065	0.51	0.002	0.012	0.85
rs808686	ascorbate	20	19717135	*AL121761.1*	A	G	0.49	21.83	0.060	0.010	3.01E-06	0.019	0.020	0.36	-0.002	0.004	0.64
rs8105491	ascorbate	19	20403988	*CTC-260E6.6*	T	G	0.85	22.24	-0.070	0.010	2.30E-06	-0.051	0.027	0.06	0.006	0.005	0.26
rs9419004	ascorbate	10	134914522	*ADGRA1*	C	G	0.28	20.35	-0.250	0.060	6.53E-06	0.057	0.030	0.06	-0.003	0.005	0.49
rs9606290	ascorbate	22	20218237	*MYO18B*	A	G	0.26	20.40	0.160	0.040	6.32E-06	0.068	0.026	0.01	-0.006	0.005	0.20
rs10019071	retinol	4	181442264	*RN7SKP13*	A	G	0.01	16.72	0.660	0.160	3.64E-06	0.149	0.104	0.15	-0.004	0.015	0.77
rs112293959	retinol	15	80358561	*ZFAND6*	G	A	0.99	10.88	-0.430	0.130	5.70E-06	0.091	0.061	0.13	0.006	0.011	0.57
rs114515641	retinol	3	118837344	*IGSF11*	G	T	0.03	10.32	0.410	0.130	7.12E-06	-0.023	0.055	0.68	0.015	0.011	0.18
rs1153379	retinol	21	16678993	*AP000472.3*	A	G	0.95	14.97	-0.320	0.080	6.10E-06	-0.045	0.067	0.50	-0.014	0.009	0.09
rs1176744	retinol	11	113803028	*HTR3B*	C	A	0.31	20.97	-0.210	0.050	3.52E-07	-0.040	0.021	0.05	-0.010	0.004	0.02
rs118025446	retinol	6	84904002	*CEP162*	A	G	0.04	17.38	-0.480	0.120	9.84E-06	-0.050	0.053	0.35	-0.019	0.010	0.05
rs12955464	retinol	18	10141045	*RP11-419J16.1*	G	C	0.87	14.79	-0.230	0.060	3.71E-06	-0.075	0.039	0.05	-0.008	0.006	0.16
rs139726207	retinol	2	223878661	*SERPINE2*	G	A	0.91	11.21	0.370	0.110	4.46E-06	0.037	0.036	0.30	NA	NA	NA
rs149113848	retinol	2	81021743	*AC084193.1*	G	C	0.99	13.21	-0.960	0.270	3.47E-06	NA	NA	NA	-0.011	0.025	0.67
rs149478645	retinol	7	117400313	*CTTNBP2*	G	A	0.97	12.79	-0.510	0.140	1.30E-06	0.064	0.088	0.47	-0.022	0.014	0.11
rs17005512	retinol	4	83113756	*RP11-576N17.4*	C	G	0.17	13.33	-0.220	0.060	2.77E-06	0.010	0.021	0.63	-0.004	0.005	0.51
rs1842947	retinol	17	3316976	*OR3A3*	G	A	0.49	20.06	-0.190	0.040	8.34E-07	-0.010	0.021	0.63	0.003	0.004	0.47
rs2147337	retinol	20	1612282	*SIRPG*	G	T	0.66	12.68	0.160	0.040	9.00E-06	-0.002	0.020	0.91	0.004	0.004	0.29
rs2367816	retinol	2	33873296	*HNRNPA1P61*	G	A	0.26	19.64	0.230	0.050	9.46E-06	0.028	0.022	0.21	-0.001	0.005	0.87
rs2417325	retinol	12	14171015	*GRIN2B*	T	C	0.09	15.49	0.330	0.080	1.29E-06	0.001	0.037	0.99	-0.007	0.007	0.32
rs3890033	retinol	3	195591527	*TNK2*	C	T	0.50	11.76	0.140	0.040	8.56E-06	0.067	0.026	0.01	NA	NA	NA
rs3898702	retinol	3	8011098	*LMCD1-AS1*	T	C	0.21	16.35	-0.220	0.050	3.02E-06	0.011	0.025	0.66	-0.004	0.005	0.41

Online Supporting Material

rs4135385	retinol	3	41279440	CTNNB1	G	A	0.76	18.75	0.210	0.050	9.80E-06	0.029	0.023	0.20	0.010	0.005	0.02
rs58411567	retinol	12	124387574	DNAH10	A	G	0.25	15.92	-0.210	0.050	3.02E-07	-0.032	0.023	0.16	0.002	0.005	0.68
rs6550239	retinol	3	20001318	RAB5A	A	G	0.73	14.51	-0.180	0.050	4.40E-06	0.026	0.023	0.24	-0.006	0.005	0.19
rs75308833	retinol	5	34602282	CTD-2197M16.1	T	C	0.97	11.27	-0.490	0.150	3.51E-06	-0.028	0.067	0.68	-0.005	0.012	0.69
rs7926028	retinol	11	50213631	RP11-347H15.6	T	G	0.47	10.39	-0.130	0.040	2.75E-06	-0.008	0.021	0.69	NA	NA	NA
rs945817	retinol	6	75454012	RNU6-248P	A	G	0.83	25.32	-0.280	0.050	6.46E-07	-0.010	0.024	0.67	-0.001	0.005	0.88
rs9586119	retinol	13	88613487	TET1P1	C	T	0.11	18.24	0.350	0.080	3.34E-06	0.036	0.036	0.32	0.011	0.007	0.11

Abbreviations: Chr, Chromosome; EA, effect allele; EAF, effect allele frequency; NA, not available; OA, other allele; Pos, position; SE, standard error; SNP, single nucleotide polymorphism.

The unit of beta coefficients is log-odds per allele.

Online Supporting Material

Supplemental Table S6: IVW findings and sensitivity analyses for genetically predicted per unit increase in circulating antioxidants and parental lifespan outcome.

Antioxidants	IVW/Wald ratio			Weighted Median		MR-Egger			MR-PRESSO		
	Beta (95% CI)	*P value*	*P value for heterogeneity*	Beta (95% CI)	*P value*	Beta (95% CI)	*P value*	Intercept (*P value*)	No. of outlier	Beta (95% CI)	*P value*
**Absolute circulating levels**											
Ascorbate (μmol/L)	-0.05 (-0.49-0.39)	0.825	0.285	0.10 (-0.42-0.61)	0.708	0.19 (-0.51-0.89)	0.607	-0.002 (0.409)	0	-0.050 (-0.49-0.39)	0.830
Lycopene (μg/dL)	0.07 (-0.15-0.29)	0.521	0.114	0.11 (-0.11-0.33)	0.330	1.00 (0.95-1.05)	0.953	0.003 (0.689)	0	0.07 (-0.15-0.29)	0.556
Selenium (ln-transformed)	-0.24 (-0.51-0.04)	0.098	0.183	-0.23 (-0.49-0.02)	0.075	-0.21 (-1.32-0.91)	0.751	-0.000 (0.963)	0	-0.24 (-0.51-0.04)	0.196
β-carotene (ln-transformed)	0.30 (-0.06-0.66)	0.098	0.680	0.29 (-0.11-0.68)	0.161	-0.08 (-1.43-1.28)	0.930	0.005 (0.551)	NA	NA	NA
Retinol (ln-transformed)	0.00 (-1.83-1.84)	0.997	0.347	NA	NA	NA	NA	NA	NA	NA	NA
**Antioxidant metabolites**											
α-tocopherol (log10 units)	0.08 (-1.29-1.45)	0.906	0.514	0.83 (-1.02-2.67)	0.380	-1.15 (-4.05-1.75)	0.460	0.003 (0.352)	0	0.08 (-1.22-1.39)	0.904
γ-tocopherol (log10 units)	-0.62 (-1.57-0.34)	0.205	0.102	-0.03 (-1.11-1.05)	0.956	0.78 (-1.09-2.66)	0.432	-0.005 (0.128)	0	-0.62 (-1.57-0.34)	0.231
Retinol (log10 units)	0.17 (0.07-0.27)	**0.001**	0.393	0.14 (0.00-0.29)	**0.048**	0.13 (-0.16-0.42)	0.395	0.001 (0.794)	0	0.17 (0.07-0.27)	**0.003**
Ascorbate (log10 units)	-0.03 (-0.27-0.22)	0.817	0.792	0.12 (-0.25-0.48)	0.533	0.18 (-0.26-0.62)	0.430	-0.002 (0.174)	0	-0.03 (-0.23-0.17)	0.782

Abbreviations: IVW, Inverse-Variance weighted; MR-PRESSO, MR Pleiotropy RESidual Sum and Outlier; NA, not available.

Online Supporting Material

Supplemental table S7: Details of GWAS datasets used to test associations of antioxidant-instrumenting SNPs with potential confounders and other determinants of longevity.

Trait	PMID	Access in MR-Base	N	Ethnicity	Absolute antioxidants SNPs available in datasets					Antioxidant metabolites SNPs available in datasets			
					ascorbate	Lycopene	Selenium	β-carotene	retinol	α-tocopherol	γ-tocopherol	Ascorbate	Retinol
Education[1]	27225129	ieu-a-1001	1,196	European	10	5	4	3	2	11	13	14	24
Smoking initiation[2]	20418890	ieu-a-962	1,232,091	European	6[3]	4[5]	4	3	2	11	13	13[6]	15[7]
T2DM	22885922	ieu-a-26	898,130	European	5[4]	5	4	3	2	11	13	14	15[7]
BMI	25673413	ieu-a-835	~700,000	European	6[3]	5	4	3	2	11	13	14	15[7]
LDL-c	27005778	met-c-895	188,577	primarily European	10	5	4	3	2	11	13	14	24
HDL-c	27005778	met-c-864	188,577	Primarily European	10	5	4	3	2	11	13	14	24
Hypertension	-	UKB-b:12493	>1million	European	10	5	4	3	2	11	13	14	24

[1] Number of years of schooling completed.

[2] Ever vs. never being a regular smoker.

[3] rs10051765, rs117885456, rs2559850, and rs6693447 were missing and no suitable proxy was found in smoking initiation dataset.

[4] rs10051765, rs117885456, rs2559850, rs33972313, and rs6693447 were missing and no suitable proxy was found in smoking initiation dataset.

[5] rs2232315 was missing and no suitable proxy was found in smoking initiation dataset.

[6] rs9419004 was missing and no suitable proxy was found in smoking initiation dataset.

[7] rs112293959, rs114515641, rs1153379, rs12955464, rs149113848, rs149478645, rs3898702, rs58411567, rs75308833 was missing and no suitable proxy was found in these outcome datasets

Abbreviations: BMI, body mass index; HDL-c, high-density lipoprotein cholesterol; LDL-c, low-density lipoprotein cholesterol; SNPs, single-nucleotide polymorphisms; T2DM, type 2 diabetes mellitus.

Online Supporting Material

Supplemental table S8: Comparison of the impacts on circulating antioxidant levels between genetic instruments and antioxidant supplementation.

Antioxidants	Genetic instruments		Antioxidant supplementation	
	Fold or concentration change	Expose duration	Fold or concentration change	Expose duration
Ascorbate	1.14-fold in μmol/L[a] [1]	Life-long (presumed)	1.15-14 fold in μmol/L	0.23-10.1 years
Lycopene	0.16 μmol/L[b] [2]		0.11-0.57 μmol/L	2-8 weeks
Selenium	0.95-1.03 μg/g per effect allele[3]		0.7-1.6 μmol/L	0.25-9 years
β-carotene	2.1 to 2.5-fold in nmol/L[c] [4]		0.60 to 10.11-fold in mg/dL	0.5-12 years
Retinol	1.13 to 1.15-fold in μg/L[d] [5]		0.86 to 1.06 fold in ug/L	0.003-6 years

a. Q5 vitamin C-raising genetic risk score compared with Q1.

b. A allele in rs7680948 was related to 8.6 ug/dL decreased serum lycopene level; for other 4 SNPs included in this MR study (rs4635297, rs341075, rs6108801, rs2232315), the effect of genetic variants on circulating lycopene was not reported in original publication.

c. Based on rs12934922 and rs7501331 only.

d. Two copies of both variant alleles (rs1667255: C/C, and rs10882272: C/C) related to 12.7–15.1% higher circulating retinol level.

Online Supporting Material

Reference:

1. Zheng, J.-S.; Luan, J.a.; Sofianopoulou, E.; Imamura, F.; Stewart, I.D.; Day, F.R.; Pietzner, M.; Wheeler, E.; Lotta, L.A.; Gundersen, T.E., et al. Plasma Vitamin C and Type 2 Diabetes: Genome-Wide Association Study and Mendelian Randomization Analysis in European Populations. *Diabetes Care* **2021**, *44*, doi:10.2337/dc20-1328.
2. D'Adamo, C.R.; D'Urso, A.; Ryan, K.A.; Yerges-Armstrong, L.M.; Semba, R.D.; Steinle, N.I.; Mitchell, B.D.; Shuldiner, A.R.; McArdle, P.F. A Common Variant in the SETD7 Gene Predicts Serum Lycopene Concentrations. *Nutrients* **2016**, *8*, 82, doi:10.3390/nu8020082.
3. Cornelis, M.C.; Fornage, M.; Foy, M.; Xun, P.; Gladyshev, V.N.; Morris, S.; Chasman, D.I.; Hu, F.B.; Rimm, E.B.; Kraft, P., et al. Genome-wide association study of selenium concentrations. *Hum Mol Genet* **2015**, *24*, 1469-1477, doi:10.1093/hmg/ddu546.
4. Leung, W.C.; Hessel, S.; Méplan, C.; Flint, J.; Oberhauser, V.; Tourniaire, F.; Hesketh, J.E.; von Lintig, J.; Lietz, G. Two common single nucleotide polymorphisms in the gene encoding β-carotene 15,15′-monoxygenase alter β-carotene metabolism in female volunteers. *FASEB J.* **2009**, *23*, 1041-1053, doi:10.1096/fj.08-121962.
5. Mondul, A.M.; Yu, K.; Wheeler, W.; Zhang, H.; Weinstein, S.J.; Major, J.M.; Cornelis, M.C.; Mannisto, S.; Hazra, A.; Hsing, A.W., et al. Genome-wide association study of circulating retinol levels. *Hum Mol Genet* **2011**, *20*, 4724-4731, doi:10.1093/hmg/ddr387.
